# Tropane alkaloids biosynthesis involves an unusual type III polyketide synthase and non-enzymatic condensation

**DOI:** 10.1038/s41467-019-11987-z

**Published:** 2019-09-06

**Authors:** Jian-Ping Huang, Chengli Fang, Xiaoyan Ma, Li Wang, Jing Yang, Jianying Luo, Yijun Yan, Yu Zhang, Sheng-Xiong Huang

**Affiliations:** 1 State Key Laboratory of Phytochemistry and Plant Resources in West China, and CAS Center for Excellence in Molecular Plant Sciences, Kunming Institute of Botany, Chinese Academy of Sciences, Kunming, 650201 China; 2 The Key Laboratory of Synthetic Biology, and CAS Center for Excellence in Molecular Plant Sciences, Institute of Plant Physiology and Ecology, Chinese Academy of Sciences, Shanghai, 200032 China; 3 School of Chemistry and Chemical Engineering, Sichuan University of Science & Engineering, Zigong, 643000 China

**Keywords:** Structural biology, Secondary metabolism, X-ray crystallography

## Abstract

The skeleton of tropane alkaloids is derived from ornithine-derived *N*-methylpyrrolinium and two malonyl-CoA units. The enzymatic mechanism that connects *N*-methylpyrrolinium and malonyl-CoA units remains unknown. Here, we report the characterization of three pyrrolidine ketide synthases (PYKS), *Aa*PYKS, *Ds*PYKS, and *Ab*PYKS, from three different hyoscyamine- and scopolamine-producing plants. By examining the crystal structure and biochemical activity of *Aa*PYKS, we show that the reaction mechanism involves PYKS-mediated malonyl-CoA condensation to generate a 3-oxo-glutaric acid intermediate that can undergo non-enzymatic Mannich-like condensation with *N*-methylpyrrolinium to yield the racemic 4-(1-methyl-2-pyrrolidinyl)-3-oxobutanoic acid. This study therefore provides a long sought-after biosynthetic mechanism to explain condensation between *N*-methylpyrrolinium and acetate units and, more importantly, identifies an unusual plant type III polyketide synthase that can only catalyze one round of malonyl-CoA condensation.

## Abstract (web summary)

Tropane alkaloids are medicinally significant plant metabolites generated by an unusual condensation of ornithine-derived and malonyl-CoA precursors. Here, Huang et al. show that pyrrolidine ketide synthase catalyzes a single round of condensation, which is followed by a non-enzymatic Mannich-like condensation.

## Introduction

**Fig. 1:**
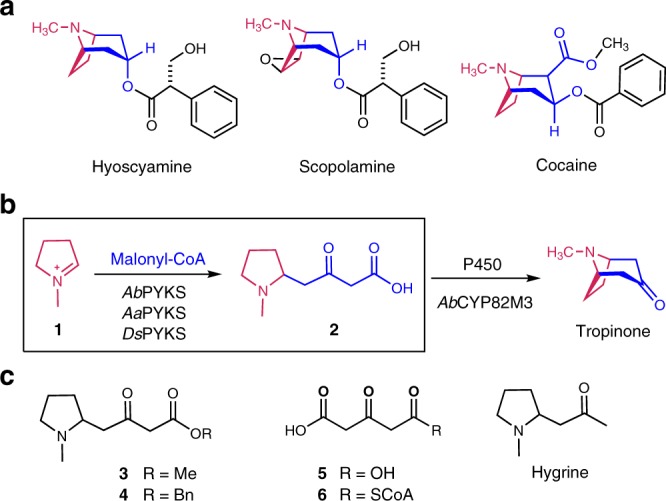
The chemical structures mentioned in this study and the biosynthetic pathway of tropinone. **a** The tropane alkaloids. **b** The tropinone biosynthesis pathway with the current study highlighted in the box. **c** The chemical structures of other compounds

Tropane alkaloids (TA) are an important class of secondary metabolites with a characteristic 8-azabicyclo[3.2.1]octane ring in their chemical structures, and occur mainly in many members of the plant families Erythroxylaceae and Solanaceae^1,2^. Among the >200 known TA, the anticholinergic drugs atropine (the racemic hyoscyamine), and scopolamine as well as the stimulant cocaine (Fig. 1a) are most prominent and have been used for a long time as medicinals worldwide^2,3^.

The carbon atom source of TA is known from the amino acid ornithine^4–7^ and acetate units^8–10^ for a long time by elaborate isotope-labeling experiments. In recent 20 years, biochemical studies have demonstrated that the intermediate *N*-methylpyrrolinium cation (**1**) was biosynthesized from amino acid ornithine by three enzymes, the ornithine decarboxylase (ODC)^11^, putrescine *N*-methyltransferase^12,13^, and *N*-methylputrescine oxidase (MPO)^14–16^ (Fig. 1b). Very recently, tropinone biosynthesis has been investigated^17^, and it has been shown that the key intermediate 4-(1-methyl-2-pyrrolidinyl)-3-oxobutanoic acid (**2**)^18^ results from an atypical type III polyketide synthase (PKS) *Ab*PYKS catalyzed condensation between **1** and malonyl-CoA, whereas tropinone is formed by a P450 enzyme *Ab*CYP82M3-mediated oxidation and cyclization of racemic **2**. However, this very unusual PYKS-mediated condensation mechanism between **1** and acetate units still remains unknown. Herein, we report the characterization of three PYKSs, *Aa*PYKS, *Ds*PYKS, and *Ab*PYKS, from three respective plants, *Anisodus acutangulus*, *Datura stramonium*, and *Atropa belladonna* as well as the condensation mechanism based on the crystal structures of *Aa*PYKS.

## Results

### Identification of type III PKS genes from three solanaceous plants

The hyoscyamine and scopolamine are found in a variety of solanaceous plants including *A*. *acutangulus*, *A*. *belladonna*, and *D*. *stramonium*^1,2,19^. Their biosynthesis occurs in the plant roots^20,21^, which provides the advantage to study the biosynthesis using the plant hairy roots culture^22^. To facilitate gene discovery efforts, we constructed the hairy roots of *A*. *acutangulus*, *A*. *belladonna*, and *D*. *stramonium*, and confirmed the production of hyoscyamine and scopolamine by HPLC analysis (Supplementary Fig. 1). Previous labeling studies^4–10,23–25^ demonstrated that the second ring of TA originates from two acetate units indicating the involvement of PKS. De novo transcriptome assembly of three established hairy roots was generated using the Hiseq platform. By a combination of direct screening of the transcriptome assembly annotations, BLAST searches and rapid amplification of cDNA ends, full-length transcripts of four, five, and two putative type III PKS unigenes were obtained from three hairy roots, respectively (Supplementary Table 1). Subsequent phylogenetic tree (Supplementary Fig. 2) and amino-acid sequence alignment (Supplementary Fig. 3) indicated that *Ab*PKS2, *Aa*PKS4, *Aa*PKS2, *Ab*PKS3, and *Ds*PKS1 from three different plants, were in a clade more divergent with others and the amino-acid sequence of *Ab*PKS3 is identical with the reported *Ab*PYKS^17^. This information suggests that the five PKSs are presumably involved in TA biosynthesis.

### Determining the enzyme activity of putative PYKS

**Fig. 2:**
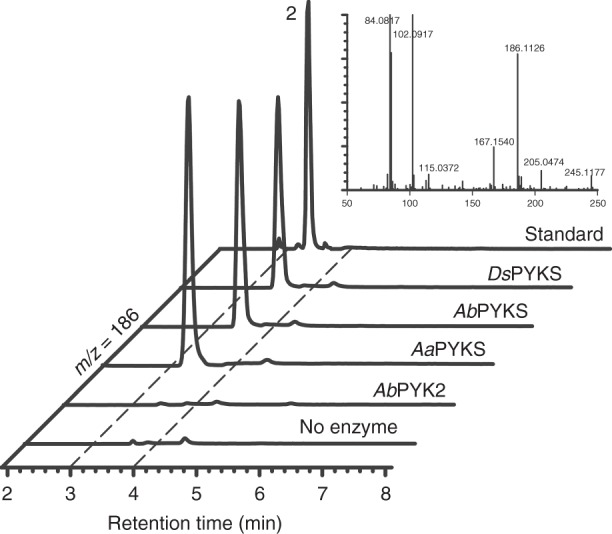
LC-MS chromatograms at [M + H]^+^ = 186 of product **2** in enzymatic reactions for *Ds*PYKS, *Ab*PYKS, *Aa*PYKS or *Ab*PKS2 using **1** and malonyl-CoA as substrates

In order to functionally assign these putative type III PKSs, the five proteins were heterologously expressed in *Escherichia coli* (*E*. *coli*) (Supplementary Fig. 4) and analyzed in vitro for their activity toward putative substrates, chemically synthesized **1** (Supplementary Fig. 5) and malonyl-CoA. Interestingly, liquid chromatography–mass spectrometry (LC-MS) analysis showed the appearance of a new peak at *m*/*z* 186 in the *Aa*PKS2, *Ab*PYKS, and *Ds*PKS1 catalyzed reactions that was not present in *Ab*PKS2 (Fig. 2 and Supplementary Fig. 6a) (*Aa*PKS4 forms inclusion bodies and was thus not characterized further). This new peak has the same MS and retention time as that of chemically synthesized **2** (Fig. 2 and Supplementary Fig. 7). Therefore, we established that *Aa*PKS2 and *Ds*PKS1 are involved in TA biosynthesis and named them as *Aa*PYKS and *Ds*PYKS, respectively^17^. However, the recent Barry and co-workers’ work did not determine the absolute configuration of the enzymatic product **2** owing to its inherent instability^17,18^. To solve this problem, we scaled up the enzymatic reactions and treated the product **2** with SOCl_2_ either in methanol or benzyl alcohol to afford the expected esters (**3** and **4**) (Fig. 1c and Supplementary Fig. 8). Then, the chiral and racemic **3** and **4** were chemically synthesized as standards for comparisons (Supplementary Fig. 8). LC-MS analysis revealed both the methyl and the benzyl product of **2** matched the standards **3** and **4** (Supplementary Fig. 9). Whereas, the optical rotation data of enzymatic product **4** is around −4.5 comparing to the value of −31.2 for synthesized *S*-configuration **4** (Supplementary Fig. 7b), suggesting that the enzymatic product **2** is a racemate.

### Structural basis for the PYKS catalytic mechanism

**Fig. 3:**
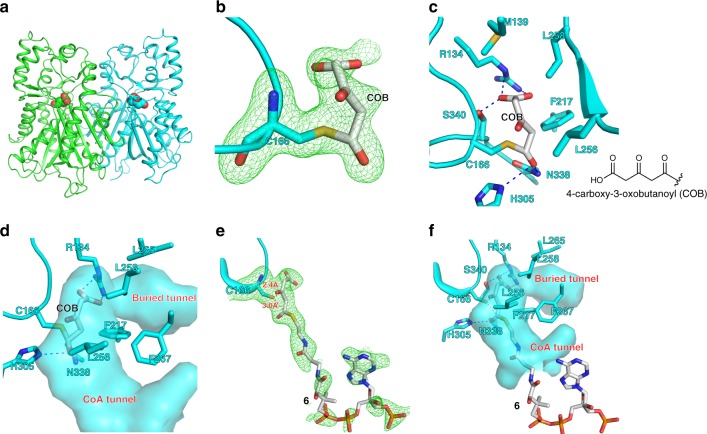
Crystal structures of *Aa*PYKS-COB (PDB ID: 6J1M) and *Aa*PYKS-**6** (PDB ID: 6J1N). **a** *Aa*PYKS-COB is a dimer (cyan and green ribbon). The 4-carboxy-3-oxobutanoyl (COB) thioester is shown as spheres. **b** The F_O_–F_C_ electron density polder map contoured at 5*σ* of the catalytic residue C166 and the covalently bound COB. **c** The detailed interactions of COB with *Aa*PYKS residues. **d** The surface presentation of *Aa*PYKS shows the inner tunnels. **e** The F_O_–F_C_ electron density polder map contoured at 3*σ* of the catalytic residue C166 and **6**. **f** The surface presentation of *Aa*PYKS-**6** shows the inner tunnels. N, O, S, and P atoms are colored as blue, red, yellow, and orange, respectively. C atoms of protein residues and small molecules are colored as cyan and white, respectively

Plant type III PKSs are a family of enzymes known to catalyze the iterative decarboxylative condensation of malonyl-CoA upon CoA-tethered substrates and could generate a variety of natural products with aromatic ring^26,27^. In this study, the type III PKSs were characterized to react onto the non-CoA-tethered substrate **1** to give a non-aromatic product, indicating a potential new family of type III PKS. To understand the structural basis for the catalytic mechanism, we determined a crystal structure of *Aa*PYKS at 2.0 Å (*Aa*PYKS-COB, Table 1; PDB ID: 6J1M). The overall structure of the dimeric *Aa*PYKS-COB is highly homologous to those of previously reported type III PKSs (Fig. 3a and Supplementary Fig. 10)^26,27^. The surface exposed characteristic CoA-binding tunnel reaches into the inner active center, where the conserved catalytic triad C166-H305-N338 resides as those in other type III PKSs. Surprisingly, we observed a large electron density extending from the thiol moiety of C166 (Fig. 3b). Inspired by previous structure of pentaketide chromone synthase (PCS) showing a co-purified CoASH in the active center^28^, we suspect that the *Aa*PYKS-COB prepared from *E*. *coli* cells probably utilized the endogenous malonyl-CoA to yield a reaction intermediate covalently bound to the thiol group. To confirm our hypothesis, we explored the possible covalent modification on C166 of purified *Aa*PYKS-COB through mass spectrometry. The results in Supplementary Fig. 11 clearly show the presence of a C166-containing peptide with an additional mass of 128 Da, corresponding to exact mass of a 4-carboxy-3-oxobutanoyl (COB) moiety (Fig. 3c). The 4-carboxy-3-oxobutanoyl thioester could be readily fit into the electron density (Fig. 3b).

**Table 1:** Data collection and refinement statistics

	*Aa*PYKS-COB	*Aa*PYKS-6
**Data collection**		
Space group	*P*3_2_21	*P*3_2_21
Cell dimensions		
*a*, *b*, *c* (Å)	108.1, 108.1, 191.1	108.4, 108.4, 191.5
α β γ (°)	90, 90, 120	90, 90, 120
Resolution (Å)	50.00–2.00 (2.03–2.00)	50.00–2.53 (2.57–2.53)
*R*_sym_ or *R*_merge_	0.104 (0.926)	0.092 (0.472)
*I*/σ*I*	24.9 (2.7)	34.1 (8.0)
Completeness (%)	100 (100)	100 (100)
Redundancy	11.0 (10.1)	16.4 (16.4)
**Refinement**		
Resolution (Å)	47.06–2.00	47.15–2.53
No. reflections	85,063	43,997
*R*_work_/*R*_free_	0.175/0.194	0.164/0.191
No. atoms	6466	6296
Protein	5805	5778
Ligand/ion	34	114
Water	627	404
*B*-factors		
Protein	27.3	32.9
Ligand/ion	39.8	63.9
Water	37.2	38.6
R.m.s. deviations		
Bond lengths (Å)	0.01	0.01
Bond angles (°)	1.14	1.45

*Values in parentheses are for highest-resolution shell

The crystal structure of *Aa*PYKS-COB also graphically explains the limited round of elongation *Aa*PYKS could catalyze. In the structure, the 4-carboxy-3-oxobutanoyl moiety is stabilized by multiple polar interactions with R134, H305, N338, and S340 and hydrophobic interactions with M139, F217, L256, and L258 (Fig. 3c). Notably, R134 and S340 make a salt-bridge and hydrogen bond interactions with the carboxy group of the thioester, respectively, which serves as a gate to prevent entry to the buried pocket near the active center, and consequently inhibits further chain elongation (Fig. 3d). Alanine, threonine, or serine substitution of R134 resulted in significant decrease of activity, highlighting the key role of R134 for *Aa*PYKS-catalyzed one-round malonyl-CoA condensation (Supplementary Fig. 6b). To explore the necessity of hydrogen bonding between S340 and the carboxy group of the thioester, amino acids without hydroxyl group (leucine, glycine, and valine) or having more steric hindrance (leucine and valine) were selected for site mutation of S340. All the three mutations showed much decreased activity (Supplementary Fig. 6c), showing the importance of hydrogen bonding to the enzymatic activity. In addition, the presence of a leucine residue in the non-functional *Ab*PKS2 at the corresponding S340 position of *Aa*PYKS also supports the key role for S340.

Our in vitro enzymatic experiment detects rapid conversion of malonyl-CoA to 3-oxo-glutaric acid (**5**) by *Aa*PYKS in the absence of **1** (Supplementary Fig. 12), implicating that malonyl-CoA could help release the covalently bound intermediate and reactivates the enzyme. We therefore soaked the crystal of *Aa*PYKS-COB with high concentration of malonyl-CoA and determined another structure at 2.5 Å (*Aa*PYKS-**6**; PDB ID: 6J1N; Table 1 and Supplementary Fig. 10b). Unexpectedly, we observed an even larger electron density than that in *Aa*PYKS-COB in the tunnel, the upper part of which is identical to that of COB moiety in the *Aa*PYKS-COB structure and the lower part of which fills up the CoA tunnel (Fig. 3e, f). The expected intermediate 4-carboxy-3-oxobutanoyl-CoA (**6**) could be readily fitted into the density. In the structure of *Aa*PYKS-**6**, the carboxyl moiety makes the same interactions as in structure of *Aa*PYKS-COB, and the sulfur of C166 is close to the C1 and C3 atoms of **6** (Fig. 3e, f). The structure suggests we trapped another intermediate **6** and the C166 is ready to attack the thiol ester carbonyl to recapture the COB moiety. To our knowledge, this is the first type III PKS structure trapped with elongated and CoA-tethered *β*-keto intermediate^29^.

The crystal structures clearly suggest that the PYKSs take the solo malonyl-CoA as both starter and extender unit. This feature is similar to that of the PCS and octaketide synthase (OKS) from *Aloe arborescens*^28,30,31^, which have been established as a novel class of plant type III PKSs because both enzymes accept the sole malonyl-CoA as both starter and extender unit to catalyze five- or eight-round malonyl-CoA condensations. Consistently, *Aa*PYKS, *Ab*PYKS, and *Ds*PYKS all have the characteristic leucine residue at 258, 259, and 259, respectively, corresponding to Leu266 in both PCS and OKS (Supplementary Fig. 3), which was proposed to determine the selectivity of the starter molecule from traditional *p*-coumaroyl-CoA to the sole malonyl-CoA unit^28,32^. Alanine substitution of *Aa*PYKS L258 led to remarkable enzymatic activity reduction (Supplementary Fig. 6). This information also suggests that all the three PKSs catalyze the sole malonyl-CoA condensation to generate **5**, then condense with **1** to yield **2** rather than **1** reacts with malonyl-CoA first (Supplementary Fig. 13). This unique order of condensations is also preferred by the feeding experiments-based TA biosynthesis by Robins et al.^23^.

### Non-enzymatic condensation 1 with 5

To determine how the non-CoA-tethered substrate **1** interacts with the enzyme, high concentration of **1** was soaked into the crystal of *Aa*PYKS-COB. However, the active center of the resulting crystal structure was highly similar to that of the structure *Aa*PYKS-COB; and the C166 is still covalently bound with the COB moiety (Supplementary Fig. 14a). This result suggests that **1** may not be capable of releasing the COB moiety from C166 in the active center. Above information in conjunction with the production of racemic **2** prompts us to propose that the condensation **1** with **5** is non-enzymatic. We next sought to obtain evidence for the non-enzymatic reactions by performing in vitro reactions between the intermediate **5** and **1** in the conditions of with and without enzyme *Aa*PYKS, respectively. As expected, both conditions did yield the same product **2**, and the kinetic analysis showed that there is no reaction velocity difference for the conditions of with or without enzyme (Supplementary Fig. 14b). These results clearly demonstrate that the racemic **2** is afforded in a non-enzymatic Mannich-like condensation. Hygrine (Fig. 1c) is a decarboxylative product of **2** biosynthetically^17,18^. Coincidentally, all the isolated hygrine from different plants is racemic^33^, corroborating the racemic feature of **2** from spontaneous condensation in TA biosynthesis.

## Discussion

**Fig. 4:**
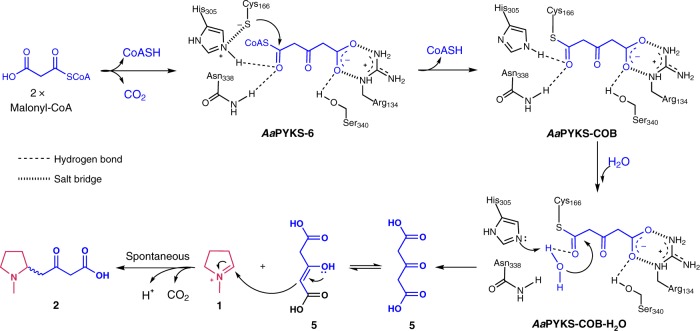
Proposed mechanism for *Aa*PYKS-catalyzed reactions

Based on the three structures and the in vitro reactions, we proposed a detailed mechanism for the identified PYKSs (Fig. 4). First, the *Aa*PYKS runs one-round malonyl-CoA condensation using conserved Cys_166_-His_305_-Asn_338_ catalytic triad to afford **6** in the active site (Fig. 3f), then the thiol group of Cys_166_ attacks the thiol ester carbonyl of **6** (Fig. 3c) to recapture the COB moiety. Subsequently one molecule of water could come into the active site to form the hydrogen bonds with His_305_ and the carbonyl as the case of the plant PKS BAS-mediated reaction^34^. The nucleophilic attack to thiol ester by this water would yield **5**. Additional malonyl-CoA will come into the active site to push **5** out of the pocket to undergo the spontaneous condensation with **1** to yield **2**.

The mechanism of Mannich-like condensation between **1** and acetate units has intrigued chemists for two decades^23–25^. Robert Robinson finished the chemical synthesis of tropinone in one pot by the addition of succinaldehyde, methylamine, and salt of 3-oxo-glutaric acid (**5**) at physiological pH condition with the 42% yield in 1917 (Supplementary Fig. 15)^35^, which has been widely applauded and has become the first synthesis classic. This classic synthesis route stimulated a proposal by Robinson, suggesting the biosynthesis of tropinone might occur via an analogous route involving the pyrrolidine ring moiety and **5** to furnish the tropane ring^18^. In this study, we clearly demonstrated that *Aa*PYKS only takes one-round of malonyl-CoA condensation to form free acid **5**, and then undergo the non-enzymatic Mannich-like condensation with the substrate *N*-methylpyrrolinium (**1**) to afford **2** (Fig. 4). This biosynthetic route is analogous to the Robinson’s proposal and suggests that the classic synthetic route reported > 100 years ago is biomimetic. Overall, our experiments have solved the longstanding question for reaction mechanism between **1** and acetate units in TA biosynthesis.

There is no reported type III PKS that takes one round of sole malonyl-CoA condensation in natural product biosynthesis, thereby, PYKS may represent an emerging class of plant type III PKSs. In addition, the key step of granatane alkaloids (GA)^36^ and lycopodium alkaloids^37,38^ biosynthesis was proposed to be similar with that of TA^17^. Our findings suggest a unifying biosynthetic mechanism for a wide range of plant alkaloids including granatane and lycopodium families (Supplementary Fig. 16). Therefore, the mechanism depiction could provide a molecular blueprint for generating structure diversified TA, GA, and lycopodium alkaloids.

## Methods

### General experimental procedures

All moisture or oxygen-sensitive reactions were carried out under an argon or nitrogen atmosphere in oven or heat-dried flasks. The solvents used were purified by distillation over the drying agents indicated and were transferred under argon: THF (Na), CH_2_Cl_2_ (CaH_2_), MeOH (Mg), NEt_3_ (CaH_2_). All reactions were monitored by thin-layer chromatography on silica gel F254 plates using UV light as visualizing agent (if applicable), and a solution of phosphomolybdic acid (50 g L^−1^) in EtOH followed by heating as developing agents. The products were purified by flash column chromatography on silica gel (200−300 meshes from the Anhui Liangchen Silicon Material Company in China). ^1^H NMR and ^13^C NMR spectra were recorded in D_2_O, CDCl_3_ or acetone-*d*_6_ solution on a Bruker AM 400 MHz instrument. Chemical shifts were denoted in ppm (*δ*), and calibrated by using residual undeuterated solvent D_2_O (4.79 ppm), CDCl_3_ (7.27 ppm), acetone-*d*_6_ (2.05 ppm) or tetramethylsilane (0.00 ppm) as internal reference for ^1^H NMR and the deuterated solvent CDCl_3_ (77.00 ppm), acetone-*d*_6_ (29.8 ppm) or tetramethylsilane (0.00 ppm) as internal standard for ^13^C NMR. The following abbreviations were used to explain the multiplicities: s = singlet, d = doublet, t = triplet, q = quartet, br = broad, brs = broad singlet, m = multiplet. Optical rotation data was measured on a Rudolph Autopol VI Automatic Polarimeter. The high-resolution mass spectral analysis (HRESIMS) data were measured on Agilent G6230 Q-TOF mass instrument (Agilent Corp., USA) by means of the ESI technique. LC-MS analysis was conducted on AGILENT 1290/6530 system. HPLC analysis was conducted on a HITACHI Chromaster system equipped with a DAD detector, a YMC-Triart C_18_ column (250 mm × 4.6 mm i.d., 5 μm), and a flow rate of 1.0 mL min^−1^ at a column temperature of 25 °C.

### Plant materials

The seeds of *A*. *acutangulus*, *A*. *belladonna*, and *D*. *stramonium* were collected from Yunnan province, Hunan province, and Heilongjiang province of China, respectively. The hairy root induction was performed using young plant tissues^39^. In brief, leaf disks and stem segments prepared from 2–6-week-old sterilized plant seedlings were inoculated with *Agrobacterium tumefaciens* strain C58C1 (pRiA4) suspended in liquid Murashige and Skoog (MS) medium containing 30 g L^−1^ sucrose and 100 µm acetosyringone, incubated in darkness on co-cultivation solid ½ MS medium containing 30 g L^−1^ sucrose and 100 µm acetosyringone, disinfected on solid ½ MS medium containing 30 g L^−1^ sucrose and 0.5 g L^−1^ cefotaxime, and sub-cultured in solid or liquid ½ MS medium with 30 g L^−1^ sucrose.

### Scopolamine and hyoscyamine analysis

Scopolamine and hyoscyamine were extracted from 40 °C dried hairy roots^40^. An extraction solvent of chloroform: methanol: 25% ammonia (15:5:1, v/v/v; 100 mL g^−1^ sample) was added to the weighed sample (200 mg), vortexed, and sonicated for 30 min, and then centrifuged for 30 min at room temperature (rt). The supernatant was evaporated to dryness at 40 °C, and the resulting sample was dissolved in 5 mL of 0.5 m sulfuric acid and 15 mL chloroform. The sulfuric acid phase was collected and adjusted to pH ~ 10 with ice-bathed 25% ammonia, and then alkaloids were extracted from the sulfuric acid phase with 5 mL of chloroform thrice. The combined chloroform phase was evaporated to dryness at 40 °C, and the resulting sample was dissolved in 1 mL methanol for HPLC analysis. The mobile phase was made up from 57% or 59% HPLC gradient methanol and 43% or 41% HPLC gradient water (containing 50 mm ammonium acetate and adjust pH to 4.6 by acetic acid). The chromatogram was monitored by detecting the absorbance at 215 nm. Standards for hyoscyamine and scopolamine (Yuanye, Shanghai, China) were prepared in methanol at a final concentration of 1 mg mL^−1^. Hyoscyamine and scopolamine peak in samples were identified by comparing their retention time and UV spectra with those of standards and confirmed by MS spectra. Quantitative analysis was performed using standard curves of hyoscyamine and scopolamine based on the peak area.

### Time-course culture of hairy roots and **de novo** transcriptome sequencing

Fresh hairy roots (0.2 g) were transferred to 100 mL liquid ½ MS medium with 30 g L^−1^ sucrose and maintained in darkness at 25 °C on a rotary shaker (110 rpm.). Samples of cultured hairy roots were collected every 5 days, and the fresh weights, dry weights, and the contents of TA were determined in triplicates. To maximize recovery of genes involved in TA biosynthesis, the 5d-old hairy roots showing the highest increase and the 15d-old hairy roots achieving maximum alkaloid content were selected as transcriptome study materials based on the time course of alkaloid production. Total RNAs of the representative 5d-old and 15d-old hairy roots were extracted, and cDNA libraries were generated and sequenced on a Hiseq platform (Personalbio, Shanghai, China). De novo assemled transcriptomes of *A*. *acutangulus* hairy roots, *A*. *belladonna* hairy roots, and *D*. *stramonium* hairy roots were generated using Trinity (r20140717, K-mer 25 bp), and 229,401 unigenes, 163,301 unigenes, and 103,470 unigenes were obtained, respectively. Databases including NR, GO, KEGG, eggNOG, and SwissProt were used for unigene function annotation.

### Gene cloning of plant type III PKSs

Total RNAs were extracted from hairy roots with Total RNA isolation kit (Promega), and quantified using NanoDrop 2000C (Thermo Scientific). In all, 1 µg of total RNA was used to prepare cDNA using SMARTer RACE 5’/3’ Kit (Clontech Laboratories, Inc.). Full-length *AaPKS*s, *AbPKS*s, and *DsPK*Ss were obtained using 5’-RACE and 3’-RACE PCR with primers listed in Supplementary Table 2.

### Multiple sequence alignment and phylogenetic analysis

Sequences of *Aa*PKSs, *Ab*PKSs, *Ds*PKSs, and representative plant and bacterial type III PKSs were aligned by MUSCLE implemented in MEGA version 7.0.14^41^, and visualized by GeneDoc version 2.7. The phylogenetic tree was generated with MEGA version 7.0.14 using the Maximum Likelihood method, a bootstrap test of 2000 replicates, and the Jones-Taylor-Thornton model using default parameters.

### Protein expression and purification

The full-length sequences of *AaPYKS*, *AbPYKS*, *AbPKS2*, *DsPYKS* were amplified from hairy root cDNA respectively using the primers listed in Supplementary Table 2. The PCR products were confirmed by agarose gel electrophoresis, purified by gel extraction kit (omega, D2500–02), digested by *Eco*RI/*Sal* I (*AaPYKS*) or *Bam*HI/*Sal*I (*AbPYKS*, *AbPKS2,* and *DsPYKS*), ligated to pET28a vector digested by the corresponding restriction enzymes using T4 ligase, and transformed into chemically competent *E*. *coli* cells DH5α. Recombinant colonies were selected on LB agar plates supplemented with kanamycin (50 μg mL^−1^). Positive clones were identified by colony PCR and the corresponding plasmids were isolated and confirmed by sequencing and restriction enzyme digestion. Site mutated pET28a-*AaPYKSs* were obtained by fusion PCR using wild pET28a-*AbPYKS* as template and the primers are listed in Supplementary Table 2.

The *E*. *coli* Rosetta (DE3) cells containing pET28a-*AaPYKS*, *-AbPYKS*, *-AbPKS2*, *-DsPYKS* or site mutated *AaPYKS* were cultured in LB liquid medium supplemented with kanamycin (50 μg mL^−1^) and chloramphenicol (25 μg mL^−1^) at 37 °C in a shaker at 200 rpm. The protein expression was induced with IPTG (0.1 mm for *Ab*PYKS, *Ab*PKS2, and *Ds*PYKS; 0.5 mm for wild *Aa*PYKS and site mutated *Aa*PYKS) for 18–20 h at 16 °C when OD_600_ reached 0.6.

Cell pellets were collected at 4 °C by centrifugation and re-suspended in 50 mL ice-cold Buffer A (15 mm imidazole, 50 mm Tris, 300 mm NaCl, 10% glycerol, pH 8.0) and lysed by sonication for 4 min on ice. The supernatant was collected by centrifugation at 24,000 rpm for 30–60 min, filtered through 0.22 µm filter, and loaded onto a HisTrap FF 5 mL column. The samples were eluted by a linear imidazole gradient of buffer A and buffer B (500 mm imidazole, 50 mm Tris, 300 mm NaCl, 10% glycerol, pH 8.0). Fractions containing PKSs were concentrated to 10 mg mL^−1^ in storage buffer (100 mm NaH_2_PO_4_, 10% glycerol, pH 7.0) with Amicon Ultra-4 centrifugal filters (Ultracel, 10,000 NMWL) and stored at −80 °C.

### Synthesis of chemical compound 1

4-Aminobutyraldehydediethylacetal **s-1** (1.61 g, 10.0 mmol) was dissolved in THF (50 mL), then NEt_3_ (2.78 mL, 20.0 mmol), di*-tert-*butyldicarbonate (4.36 g, 20.0 mmol) and DMAP (37 mg, 0.3 mmol) were added. The mixture was stirred at room temperature for 18 h, and then all volatiles were removed in vacuo. The pure product was obtained by column chromatography on silica gel eluting with petroleum ether/EtOAc (5:1) to afford the compound **s-2** as a colorless oil (2.51 g, 96%)^42^.

Sodium hydride (60% dispersion in oil, 183 mg, 4.58 mmol) was added to a solution of **s-2** (1.00 g, 3.82 mmol) in DMF (5 mL). After 1 h, iodomethane (0.36 mL, 5.73 mmol) was added and the resulting mixture stirred at rt for 18 h. It was poured into water (100 mL) and extracted with EtOAc (3 × 50 mL). The organic portion was washed with brine (5 × 60 mL), dried with Na_2_SO_4_, filtered and evaporated in vacuo. The pure product was obtained by column chromatography on silica gel eluting with petroleum ether/EtOAc (5:1) to afford the compound **s-3** (0.84 g, 80%). ^1^H NMR (400 MHz, CDCl_3_): *δ* 4.45 (s, 1 H), 3.65–3.54 (m, 2 H), 3.49–3.37 (m, 2 H), 3.17 (s, 2 H), 2.19 (s, 3 H), 1.62–1.56 (m, 4 H), 1.41 (s, 9 H), 1.19–1.12 (m, 6 H); ^13^C NMR (100 MHz, CDCl_3_): *δ* 155.7, 102.5, 79.0, 61.04, 60.97, 33.9, 30.7, 28.3, 15.2 (One carbon signal for CH_2_ overlapped)^43^.

A solution of compound **s-3** (275 mg, 1.0 mmol) in MeOH (1.6 mL) was cooled in an ice bath for 10 min. A solution of HCl (4 m, 1.6 mL) was added dropwise. Then the mixture was stirred at 0 ^o^C for 20 min and then at rt for 16 h, which was used for enzymatic reaction directly. Compound **s-4:** HRESIMS (*m/z*): [M + H]^+^ calcd. for C_5_H_12_NO, 102.0913; found, 102.0915. Compound **1**: HREIMS (*m/z*): [M]^+^ calcd. for C_5_H_10_N^+^, 84.0808; found, 84.0809.

MeOH was removed in vacuo, and the aqueous layer was washed with ether to remove organic impurities. Water was evaporated under vacuum using a rotary evaporator, leaving the final compound in an oil state. ^1^H, ^13^C NMR (Supplementary Fig. 17 and 18) and high-resolution mass spectra of compound **1** were collected to confirm product identity. ^1^H NMR (400 MHz, D_2_O): *δ* 4.27–4.13 (m, 2 H), 3.63 (s, 3 H), 3.19 (brs, 2 H), 3.11–2.97 (m, 1 H), 2.39–2.29 (m, 2 H); ^13^C NMR (100 MHz, D_2_O): *δ* 181.6, 60.6, 40.4, 35.7, 19.5.

### Synthesis of chemical compound (*S*)−3

Thionyl chloride (2.61 mL, 36.0 mmol) dissolved in CHCl_3_ (15 mL) was added slowly to an ice-cold solution of (*S*)-*N*-methyl-2-(hydroxymethyl)pyrrolidine **s-5** (3.46 g, 30 mmol) in CHCl_3_ (30 mL) stirred in a N_2_ atmosphere. After the addition, the reaction mixture was stirred at rt for 2 h and then refluxed for 30 min. The solvent was then removed in vacuo and the residue dissolved in a small amount of absolute EtOH. Et_2_O was added and (*S*)-*N*-methyl-2-(chloromethyl)pyrrolidine hydrochloride **s-6** was separated (4.75 g, 93%)^9^.

(*S*)-*N*-Methyl-2-(chloromethyl)pyrrolidine hydrochloride **s-6** (4.75 g, 27.9 mmol) was dissolved in 80% aqueous EtOH (75 mL) and cooled in an ice bath. NaHCO_3_ (2.62 g, 31.2 mmol) dissolved in water (35 mL) was slowly added and the mixture stirred for 15 min. Sodium cyanide (2.05 g, 41.8 mmol) was added and the mixture refluxed for 30 min. The EtOH was then removed in vacuo and the residue extracted with EtOAc (3 × 50 mL). The dried extract (Na_2_SO_4_) was evaporated and the residual oil was chromatographed on silica gel eluting with petroleum ether/EtOAc (1:1) to afford (*S*)-*N*-methyl-2-(cyanomethyl)pyrrolidine **s-7** (2.60 g, 75% yield)^9^.

(*S*)-*N*-Methyl-2-(cyanomethyl)pyrrolidine **s-7** (620 mg, 0.50 mmol) was added to concentrated HCl (2 mL) and the mixture refluxed for 2 h. The solution was evaporated to dryness and the residue dissolved in water (1 mL). The aqueous solution was adjusted to pH 8–9 with 2 m NaOH aqueous solution and then evaporated to dryness. The residue was washed with CH_2_Cl_2_ (10 × 3 mL), the combined CH_2_Cl_2_ was dried with Na_2_SO_4_, filtered and evaporated in vacuo to give (*S*)-*N*-Methylpyrrolidine-2-acetic acid **s-8** (198 mg, 27.6%). ^1^H NMR (400 MHz, CDCl_3_, Supplementary Fig. 19): *δ* 9.90 (brs, 1 H), 3.58 (brs, 1 H), 3.21 (brs, 1 H), 2.73 (s, 3 H), 2.80–2.53 (m, 3 H), 2.28–2.17 (m, 1 H), 2.10–1.49 (m, 3 H); ^13^C NMR (100 MHz, CDCl_3_, Supplementary Fig. 20): *δ* 173.9, 64.4, 55.6, 39.4, 35.1, 30.0, 22.2.

1,1’-Carbonyldiimidazole (62.2 mg, 0.38 mmol) was taken in a 25 mL flame-dried Schlenk flask, which was under nitrogen atmosphere. The shlenk was charged with (*S*)-*N*-Methylpyrrolidine-2-acetic acid **s-8** (50.0 mg, 0.35 mmol) in THF (1 mL) and the reaction was stirred for 3 h at room temperature. Then methyl potassium malonate (81.8 mg, 0.52 mmol) and MgCl_2_ (39.8 mg, 0.42 mmol) were added to the above solution and the reaction mixture was stirred for 16 h. Water was added to the reaction mixture and extracted with EtOAc (15 mL × 3). The organic portion was dried with Na_2_SO_4_, filtered and evaporated in vacuo, and the residual oil was chromatographed on silica gel eluting with CH_2_Cl_2_/MeOH (40:1) to afford compound (*S*)**−3** as a yellow oil (24.9 mg, 35.7%). ^1^H NMR (400 MHz, acetone-*d*_6_, Supplementary Fig. 21): *δ* 3.54 (s, 3 H), 3.44 (s, 2 H), 2.86–2.74 (m, 2 H), 2.46–2.36 (m, 2 H), 2.12 (s, 3 H), 2..00 (q, *J* = 8.0 Hz, 1 H), 1.93–1.85 (m, 1 H), 1.47–1.49 (m, 2 H),1.32–1.23 (m, 1 H); ^13^C NMR (100 MHz, acetone-*d*_6_, Supplementary Fig. 22): *δ* 202.8, 168.4, 62.3, 57.3, 52.2, 50.2, 47.9, 40.6, 31.7, 22.8.

### Synthesis of chemical compounds (*S*)-4 and (*S*)-2

A solution of KOH (0.98 g, 17.6 mol) in benzyl alcohol (17.6 mL) was added gradually to a solution of dibenzyl malonate (5.00 g, 17.6 mmol) in benzyl alcohol (44 mL). The reaction mixture was stirred for 4 h at ambient temperature, and diluted with diethyl ether (250 mL), resulting in a white precipitate. The mixture was stirred for 30 min and the product was removed by filtration, washed with diethyl ether (3 × 20 mL), and air-dried to give benzyl potassium malonate as a white solid (3.34 g, 82%)^44^.

1,1’-Carbonyldiimidazole (62.2 mg, 0.38 mmol) was taken in a 25 mL flame-dried Schlenk flask, which was under nitrogen atmosphere. The shlenk was charged with (*S*)-*N*-Methylpyrrolidine-2-acetic acid **s-8** (50.0 mg, 0.35 mmol) in THF (1 mL) and the reaction was stirred for 3 h at room temperature. Then benzyl potassium malonate (121 mg, 0.52 mmol) and MgCl_2_ (39.8 mg, 0.42 mmol) were added to the above solution and the reaction mixture was stirred for 16 h. Water is added to the reaction mixture and extracted with EtOAc (15 mL × 3). The organic portion was dried with Na_2_SO_4_, filtered and evaporated in vacuo, and the residual oil was chromatographed on silica gel eluting with a mixture of CH_2_Cl_2_ and MeOH (40:1) to afford compound (*S*)**-4** as a yellow oil (32.7 mg, 34.0 %). [*α*]_D_^37^ = −31.2 (*c* 0.1, H_2_O). ^1^H NMR (400 MHz, acetone-*d*_6_, Supplementary Fig. 23): *δ* 7.35–7.12 (m, 5 H), 5.03(s, 2 H), 3.49 (s, 2 H), 2.84–2.73 (m, 2 H), 2.46–2.36 (m, 2 H), 2.10 (s, 3 H), 2.03–1.95 (m, 1 H), 1.92–1.81 (m, 1 H), 1.55–1.47 (m, 2 H), 1.30–1.20 (m, 1 H). ^13^C NMR (acetone-*d*_6_, Supplementary Fig. 24): *δ* 202.9, 168.0, 137.2, 129.4, 129.2, 129.1, 67.2, 62.5, 57.4, 50.5, 48.1, 40.8, 31.8, 23.0. HRESIMS (*m/z*): [M + H]^+^ calcd. for C_16_H_22_NO_3_, 276.1594; found, 276.1599.

A solution of the benzyl ester (*S*)**−4** (20 mg, 0.073 mmol) in methanol (2 mL) was stirred with 10% Pd/C (2 mg) under hydrogen (1 atm) at room temperature for 16 h. The mixture was then filtered through Celite and the solvent was evaporated in vacuo to give compound (*S*)**−2**, which is unstable. HRESIMS (*m/z*): [M + H]^+^ calcd. for C_9_H_16_NO_3_, 186.1125; found, 186.1124.

### Synthesis of chemical compounds 4 and 2

1 mL of **1** (in MeOH/H_2_O = 1:1) and 200 mg of **5** were added to 19 mL of potassium phosphate buffer (50 mm K_2_HPO_4_/KH_2_PO_4_, pH 8.0). The mix was stirred at 30 °C for 3 h and quenched with 20 mL of 200 mm ammonium acetate solution and 0.94 mL of formic acid (88% stock solution). Compound **2**: MS (*m/z*): [M + H]^+^; calcd. for C_9_H_16_NO_3_, 186; found, 186.

The mixture containing compound **2** was then dried by freeze dryer, and the product was further dissolved in 1 mL of benzyl alcohol. 0.1 mL of thionyl chloride was added dropwise to this solution. The resulting solution was heated at 65 °C for ~ 6 h and the crude product was the benzyl product of **2**, compound **4**. [*α*]_D_^37^ = −6.5 (*c* 0.4, H_2_O). Compound **4**: MS (*m/z*): [M + H]^+^; calcd. for C_16_H_22_NO_3_, 276; found, 276.

### Enzymatic assays and LC-MS analysis

All reactions were performed in potassium phosphate buffer (50 mm K_2_HPO_4_/KH_2_PO_4_, pH 8.0). For detection of formation of compound **2**, a reaction mixture (100 µL) containing 5 µL **1** (in MeOH/H_2_O = 1:1), 0.5 mm malonyl-CoA (Sigma-Aldrich, M4263), and 7 µm protein (*Aa*PYKS, *Ab*PYKS, *Ab*PKS2, or *Ds*PYKS) was incubated at 30 °C for 2 h. For detection of formation of compound **5**, a reaction mixture (100 µL) containing 0.5 mm malonyl-CoA and 15 µm *Aa*PYKS was incubated at 30 °C for 2 h. The reactions were stopped by addition of 100 µL ammonium acetate solution (200 mm stock solution), 4 µL formic acid (88% stock solution) and 6 µL methanol. A 10 µL aliquot of the reaction mixture was subject to LC-MS analysis eluted with a flow rate of 1 mL min^−1^ over a 25 min gradient with water and acetonitrile (*t* = 0 min, 5% acetonitrile; *t* = 10 min, 5% acetonitrile; *t* = 10.1 min, 50% acetonitrile; *t* = 20 min, 50% acetonitrile; *t* = 20.1 min, 100% acetonitrile) on a HITACHI LaChrom C8 column (4.6 mm × 250 mm, i.d., 5 µm) at 25 °C. Formic acid (final concentration, 0.1%) was added to the mobile phases for analyzing compound **5**.

Thionyl chloride (0.069 mmol, 5 µL) was added dropwise to a solution of enzymatic product **2** (estimated at 0.005 mmol, 1.0 mg) in methanol/benzyl alcohol (1 mL). The resulting solution was heated at 65 °C for ~ 6 h and the crude product was the methyl and the benzyl product of **2**, respectively.

For synthesized methyl 4-(1-methylpyrrolidinyl)-3-oxobutanoate (**3**), benzyl 4-(1-methylpyrrolidinyl)-3-oxobutanoate (**4**), and the methyl or the benzyl product of **2**, LC-MS analysis was performed on a YMC-Triant C18 column (4.6 mm I.D. × 250 mmL) at a flow rate of 1 mL min^-1^ over a 28 min gradient with water and methanol (*t* = 0 min, 10% methanol; *t* = 20 min, 100% methanol; *t* = 24 min, 100% methanol; *t* = 24.1 min, 10% methanol; *t* = 28 min, 10% methanol). The column temperature was 25 °C, and formic acid (final concentration, 0.1%) was added to the mobile phases.

For kinetic assays, potassium phosphate buffer (50 mm K_2_HPO_4_/KH_2_PO_4_, pH 8.0) containing 1 mm of compound **5** (Sigma-Aldrich, 165115–25 G) at 30 °C was added *Aa*PYKS or boiled *Aa*PYKS to 5.6 µm and the indicated amount of compound **1** to initiate the reaction (total volume was 50 µL). After 5 min incubation, the reactions were stopped and measured as described. Quantification was performed based on standard curves for synthesized methyl compound **3** and its degradation products, hygrine, and compound **2**^17^.

The large-scale enzyme reaction was performed as described in the Synthesis of chemical compounds **4** and **2** part in the present of 6 µm *Aa*PYKS. The benzyl modification product of **2** was purified by HPLC using the same gradient elution program as that for the LC-MS analysis, and its stereochemistry was studied. [*α*]_D_^37^ = −4.5 (*c* 0.40, H_2_O).

### Crystallization and structure determination of *Aa*PYKS

For preparation of *Aa*PYKS, *E*. *coli* Rosetta (DE3) cells carrying pET28a-*AaPYKS* were cultured in LB at 37 °C, and the expression of *N*-terminal 6xHis-tagged *Aa*PYKS was induced at 18 °C for 16 h with 0.5 mm IPTG at OD_600_ of 0.8. Cells were harvested by centrifugation (8000 g, 4 °C), re-suspended in lysis buffer (50 mm Tris-HCl pH 8.0, 0.3 m NaCl, 5% (v/v) glycerol, 5 mm β-mercaptoethanol, protease inhibitor cocktail (bimake.cn)) and lysed using an Avestin EmulsiFlex-C3 cell disrupter (Avestin, Inc.). The lysate was centrifuged (16,000 g; 45 min; 4 °C) and the supernatant was loaded on to a 2 mL column packed with Ni-NTA agarose (SMART, Inc.). The protein was washed by lysis buffer containing 20, 40 mm imidazole and eluted with lysis buffer containing 500 mm imidazole. The eluted fractions were loaded on a HiLoad 16/60 Superdex S200 column (GE Healthcare, Inc.) equilibrated in 10 mm Tris-HCl pH 8.0, 0.1 m NaCl, 1%(v/v) glycerol, 1 mm DTT. Fractions containing *Aa*PYKS were collected, concentrated to 15 mg mL^−1^, and stored at − 80 °C.

Crystals of *Aa*PYKS-COB were grown by vapor diffusion at 4 °C in 2 μL drops containing a 1:1 mixture of 15 mg mL^−1^ protein and crystallization buffer (1% w/v Tryptone, 0.05 m HEPES sodium 7.0, 12% PEG3,350 (w/v), 1 mm Sodium azide). *Aa*PYKS-COB crystals grown 7 days were harvested for X-ray diffraction data collection. *Aa*PYKS-**6** crystals were obtained by soaking the *Aa*PYKS-COB crystals in crystallization buffer containing 10 mm malonyl-CoA. Crystals were transferred and stabilized in crystallization buffer containing 21% ethylene glycol and cooled in liquid nitrogen.

Data were collected at Shanghai Synchrotron Radiation Facility beamlines 17U and 19U1, processed using HKL2000^45^. The structure was solved by molecular replacement with Phaser MR^46^ using the structure of chalcone synthase (PDB: 1BI5) as the search model. Cycles of iterative model building and refinement were performed in Coot^47^ and Phenix^48^.

### LC-MS/MS analysis of *Aa*PYKS

*Aa*PYKS protein (50 µg) was loaded onto a PD Minitrap G-25 column (GE Healthcare, USA) equilibrated with water to remove salt contamination. The *Aa*PYKS fractions were collected and digested with 1 µg of trypsin (Promega) in 50 µL of 50 mm NH_4_HCO_3_ for 16 h at 37 °C. The samples were subsequently evaporated to dryness. Peptides were then analyzed in an Orbitrap Fusion Lumos Mass spectrometer (Thermo Scientific, USA). Peptide fragmentation was performed via higher-energy collision dissociation. Data processing was performed using Proteome Discoverer 2.1 software (Thermo Scientific, USA) and peptide sequences were determined by matching protein databases with the acquired fragmentation pattern by SEQUEST HT algorithm. The precursor mass tolerance was set to 10 ppm and fragment ion mass tolerance to 0.05 Da.

### Reporting summary

Further information on research design is available in the Nature Research Reporting Summary linked to this article.

## Supplementary information

Supplementary Information

Reporting Summary

Source Data

## Data Availability

RNA-Seq data that support the findings of this study have been deposited in National Center for Biotechnology Information (NCBI) Sequence Read Archive (SRA) with accession numbers SRR9888534, SRR9888536, and SRR9888538. The GenBank accession numbers for *AaPYKS*, *DsPYKS*, and *AbPKS2* are MN025472, MN025473, and MN025474, respectively. The PDB IDs for AaPYKS-COB and AaPYKS-**6** are 6J1M and 6J1N, respectively. The authors declare that all other relevant data supporting the findings of this study are available within the article, its Supplementary Information, and Source Data files.
