# Supplementary material for: Tropane alkaloids biosynthesis involves an unusual type III polyketide synthase and non-enzymatic condensation
Source: Nat Commun. 2019 Sep 6;10:4036. doi: 10.1038/s41467-019-11987-z (PMC6731253; doi:10.1038/s41467-019-11987-z)
Supplement: Supplementary file 1 — Supplementary Information [file 41467_2019_11987_MOESM1_ESM.pdf]

Supplementary Figures and Tables

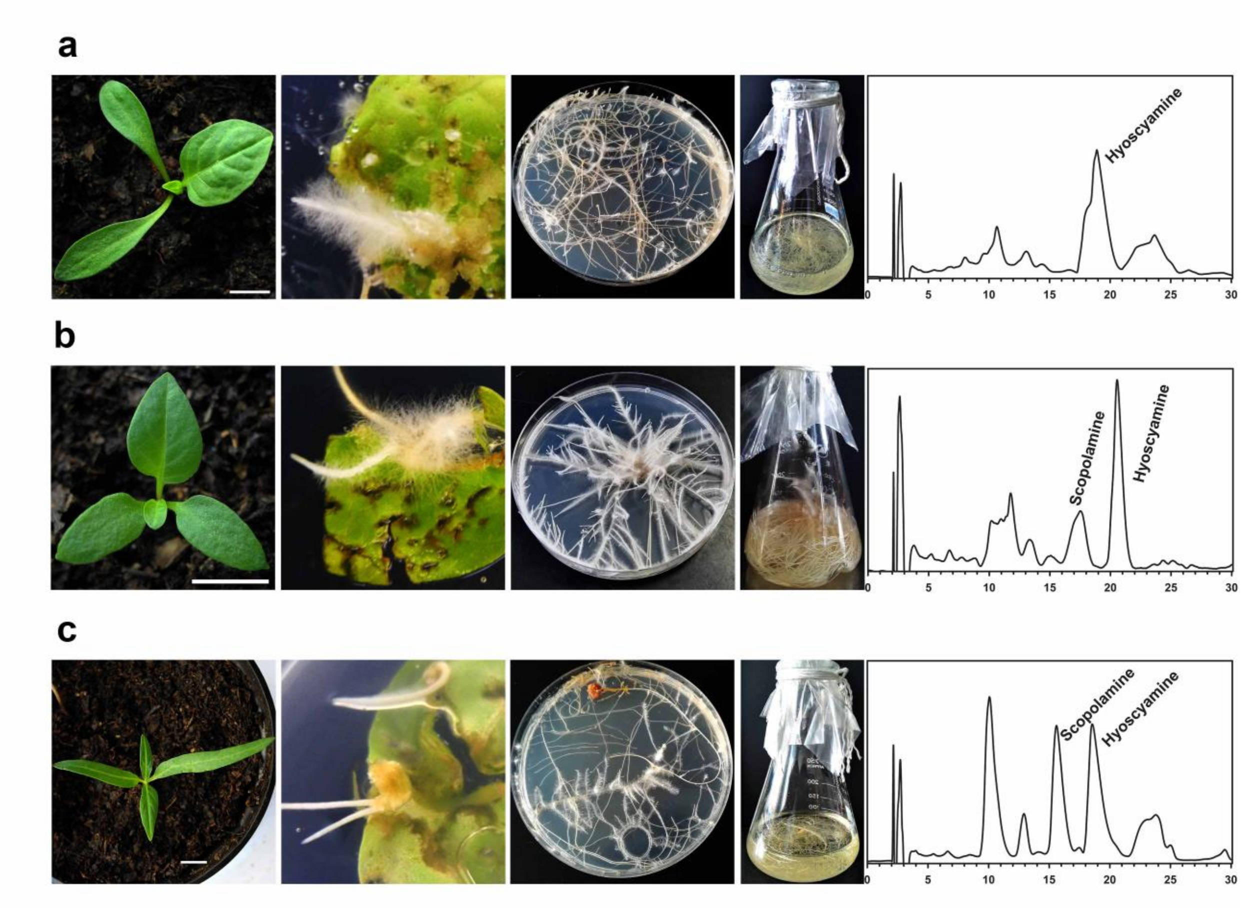

Supplementary Figure 1. Hairy roots induction of *A. acutangulus*, *A. belladonna*, and *D. stramonium*, and tropane alkaloids analysis in established culture systems by HPLC. a *A. acutangulus* (from left to right): 3-week old seedling, hairy root induced from leaf disc, hairy roots on solid medium, hairy roots in liquid medium, and tropane alkaloids in hairy roots. **b** *A. belladonna* (from left to right): 3-week old seedling, hairy root induced from leaf disc, hairy roots on solid medium, hairy roots in liquid medium, and tropane alkaloids in hairy roots. **c** *D. stramonium* (from left to right): 3-week old seedling, hairy root induced from leaf disc, hairy roots on solid medium, hairy roots in liquid medium, and tropane alkaloids in hairy roots. Scale bar = 1 cm.

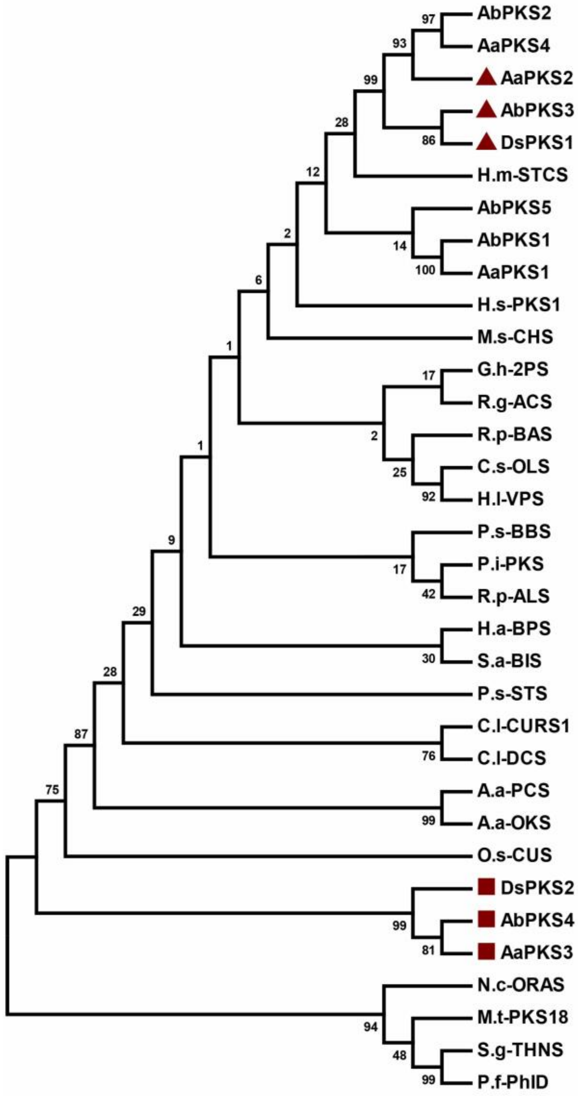

Supplementary Figure 2. Phylogenetic tree analysis of PKSs from *A. acutangulus*, *A. belladonna*, *D. stramonium*, and representative plant and bacterial type III PKSs. A.a PCS, *Aloe arborescens* (gi 60686902); C.l CURS1, *Curcuma longa* (gi 226371525); C.l DCS, *Curcuma longa* (gi 226371523); C.s OLS, *Cannabis sativa* (gi 171363647); G.h 2PS, *Gerbera hybrida* (gi 18266878); H.a BPS, *Hypericum androsaemum* (gi 18874081); H.l VPS, *Humulus lupulus* (gi 3252788); H.s PKS1, *Huperzia serrata* (gi 115338577); H.m STCS, *Hydrangea macrophylla* (gi 25988622); M.s CHS, *Medicago sativa* (gi 231782); O.s CUS, *Oryza sativa* (gi 75301441); P.i PKS, *Plumbago indica* (gi 121663842); P.s BBS, *Phalaenopsis* hybrid cultivar (gi 758243); P.s STS, *Pinus sylvestris* (sp Q02323); R.g ACS, *Ruta graveolens* (gi 10933924); R.p BAS, *Rheum palmatum* (gi 15055057); S.a BIS, *Sorbus aucuparia* (gi 82698823); R.p ALS, *Rheum palmatum* (gi 46309694); A.a OKS, *Aloe arborescens* (gi 55709867); S.g THNS, *Streptomyces griseus* (gi 3702261); P.f PhID, *Pseudomonas fluorescens* Q2-87 (AAB48106), M.t PKS18, *Mycobacterium tuberculosis* CDC1551 (gi 13881027); N.c ORAS, *Neurospora crassa* OR74A (gi 85097336). The two clades consisting of PKSs from the three TA-producing species: clade I (*Aa*PKS2, *Ab*PKS3, and *Ds*PKS1) and clade II (*Ds*PKS2, *Ab*PKS4 and *Aa*PKS3) are marked with red triangles and squares, respectively. The clade I containing the known *Ab*PKS3 (*Ab*PYKS) involved in TA biosynthesis was studied further in this paper.

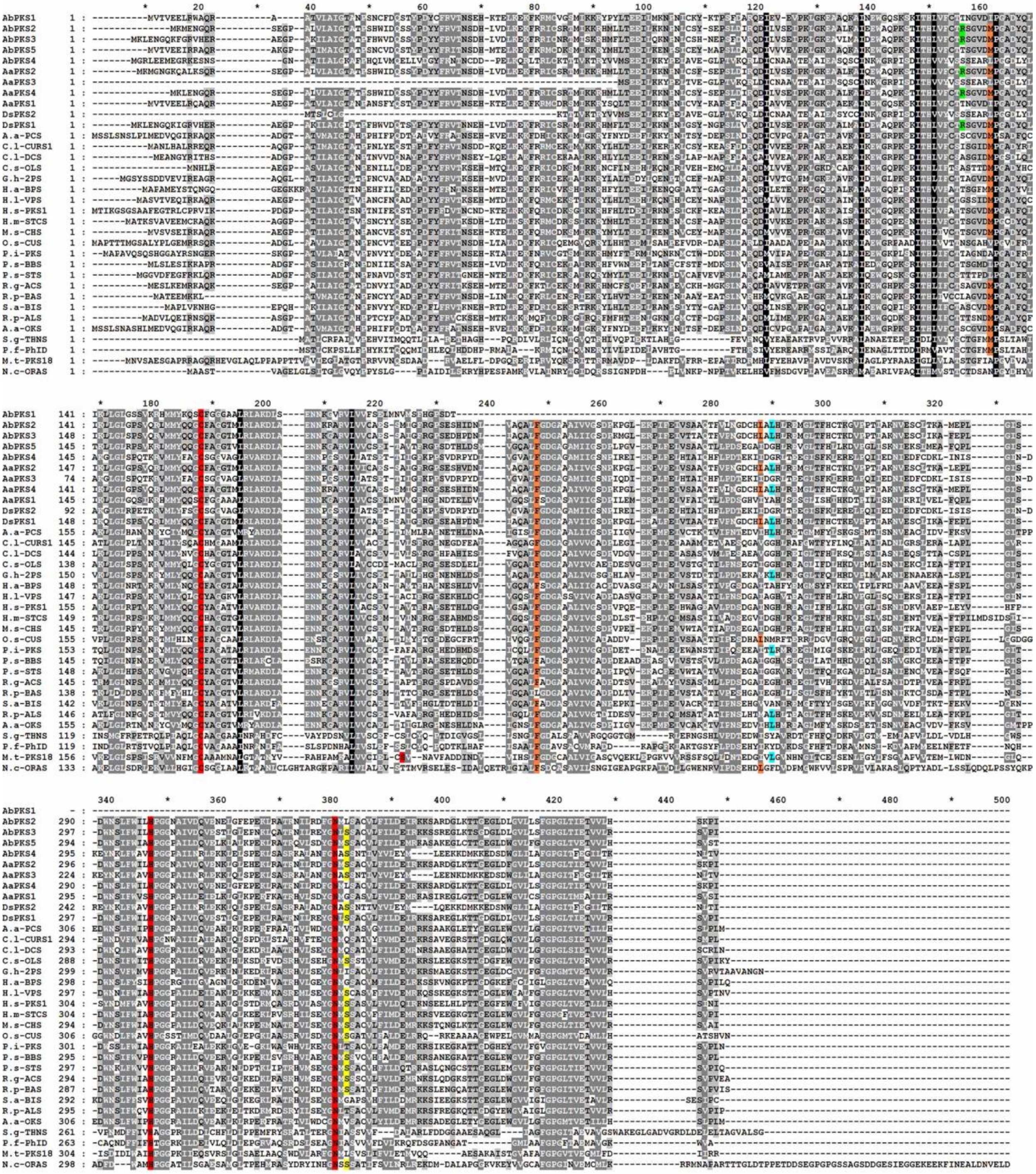

Supplementary Figure 3. Multiple sequence alignment of amino acid sequences of PKSs from *A. acutangulus*, *A. belladonna*, and *D. stramonium* and representative plant and bacterial type III PKSs. The catalytic triad Cys-His-Asn is colored in red. The leucine residues crucial for the starter molecule selection of type III PKSs are highlighted in blue. The arginine and serine residues crucial for the catalytic activity are highlighted in green and yellow, respectively. Residues involved in hydrophobic interactions are highlighted in orange except L258 which are highlighted in blue. The abbreviations are as shown in supplementary Figure 2.

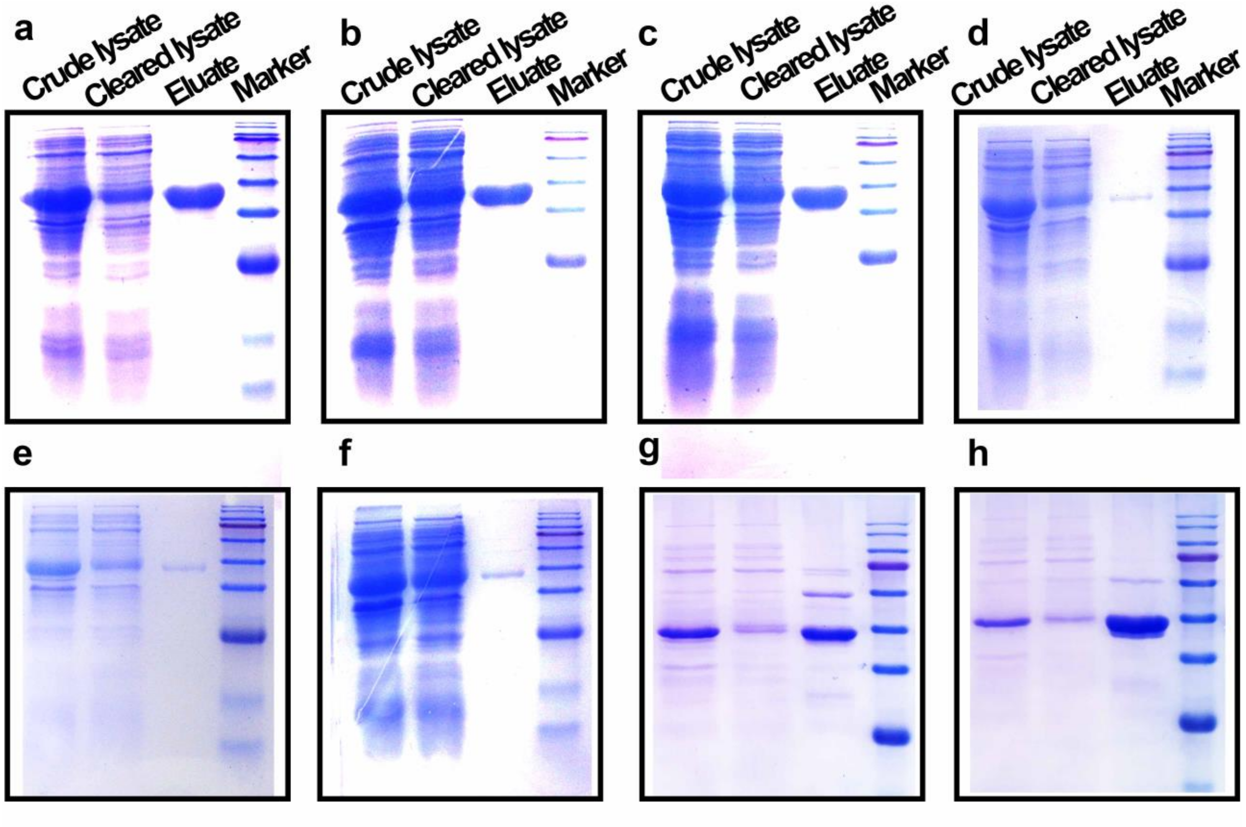

Supplementary Figure 4. SDS-PAGE analysis of the proteins purified for *in vitro* activity assays. a *Aa*PYKS. **b** *Ab*PYKS. **c** *Ds*PYKS. **d** *Aa*PYKS (R134A). **e** *Aa*PYKS (R134T). **f** *Aa*PYKS (R134S). **g** *Aa*PYKS (S340G). **h** *Aa*PYKS (L258A). Lane 1: crude lysate; lane 2: clear lysate; lane 3: elute protein; lane 4: protein molecular markers.

a

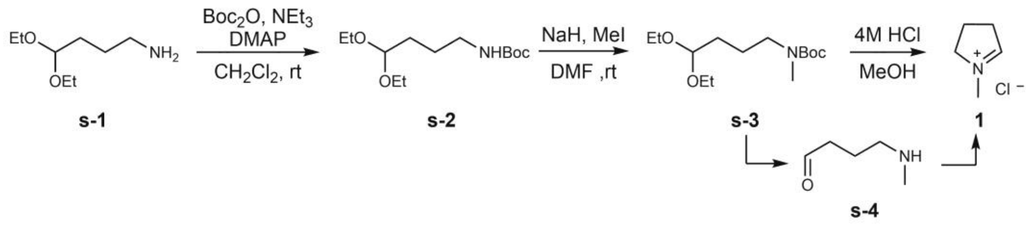

b

Qualitative Analysis Report

**Data Filename**	mxy-128.d	**Sample Name**	mxy-128
**Sample Type**	Sample	**Position**	P1-B4
**Instrument Name**	Instrument 1	**User Name**	
**Acq Method**	s.m	**Acquired Time**	12/25/2018 3:32:31 PM
**IRM Calibration Status**	Success	**DA Method**	Default.m
**Comment**			

**Sample Group**		**Info.**
**Acquisition SW**	6200 series TOF/6500 series	
**Version**	Q-TOF B.05.01 (B5125.2)	

User Spectra

Fragmentor Voltage	Collision Energy	Ionization Mode
135	0	ESI

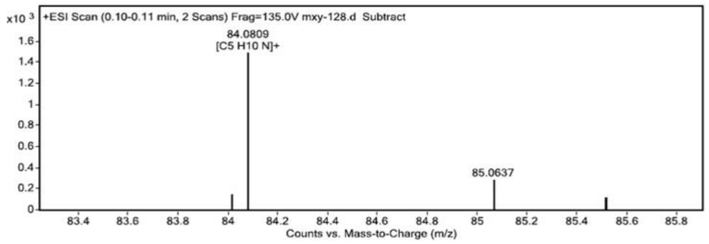

Peak List

m/z	z	Abund	Formula	Ion
61.0076		2142		
73.5321		682.96		
84.0809		1495.2	C5 H10 N	M+
86.0973		1244.47		
102.0907	1	1510.18		
110.0084		768.66		
116.1067	1	1176.32		
242.1363	1	8251.62		
243.1397	1	732.67		
258.1097		977.98		

Formula Calculator Element Limits

Element	Min	Max
C	3	60
H	0	120
O	0	30
N	0	3

Formula Calculator Results

Formula	CalculatedMass	CalculatedMz	Mz	Diff. (mDa)	Diff. (ppm)	DBE
C5 H10 N	84.0813	84.0808	84.0809	-0.10	-1.19	1.5000

--- End Of Report ---

Supplementary Figure 5. Synthesis of chemical compound 1. a The synthetic route of *N*-methylpyrrolinium cation (**1**). **b** HRMS analysis of *N*-methylpyrrolinium cation (**1**).

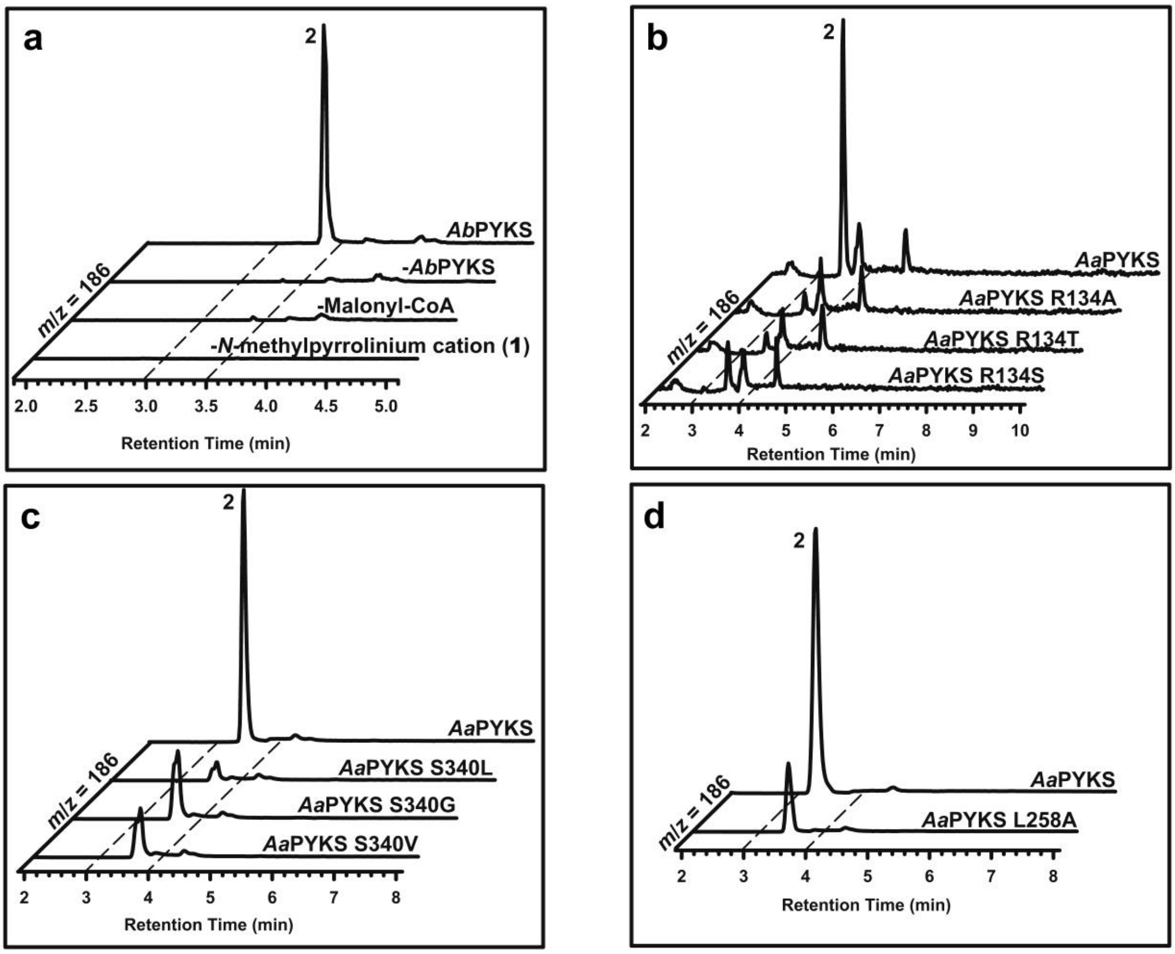

Supplementary Figure 6. LC-MS analysis of enzymatic reaction products. a LC-MS analysis of *Ab*PYKS-catalyzed enzymatic reaction products. Compound **2** ($[M + H]^+ = 186$) was obtained by using **1** and malonyl-CoA as substrates. **b, c, d** LC-MS analysis of enzymatic reaction products produced by wild-type or derivative of *Aa*PYKS using **1** and malonyl-CoA as substrates.

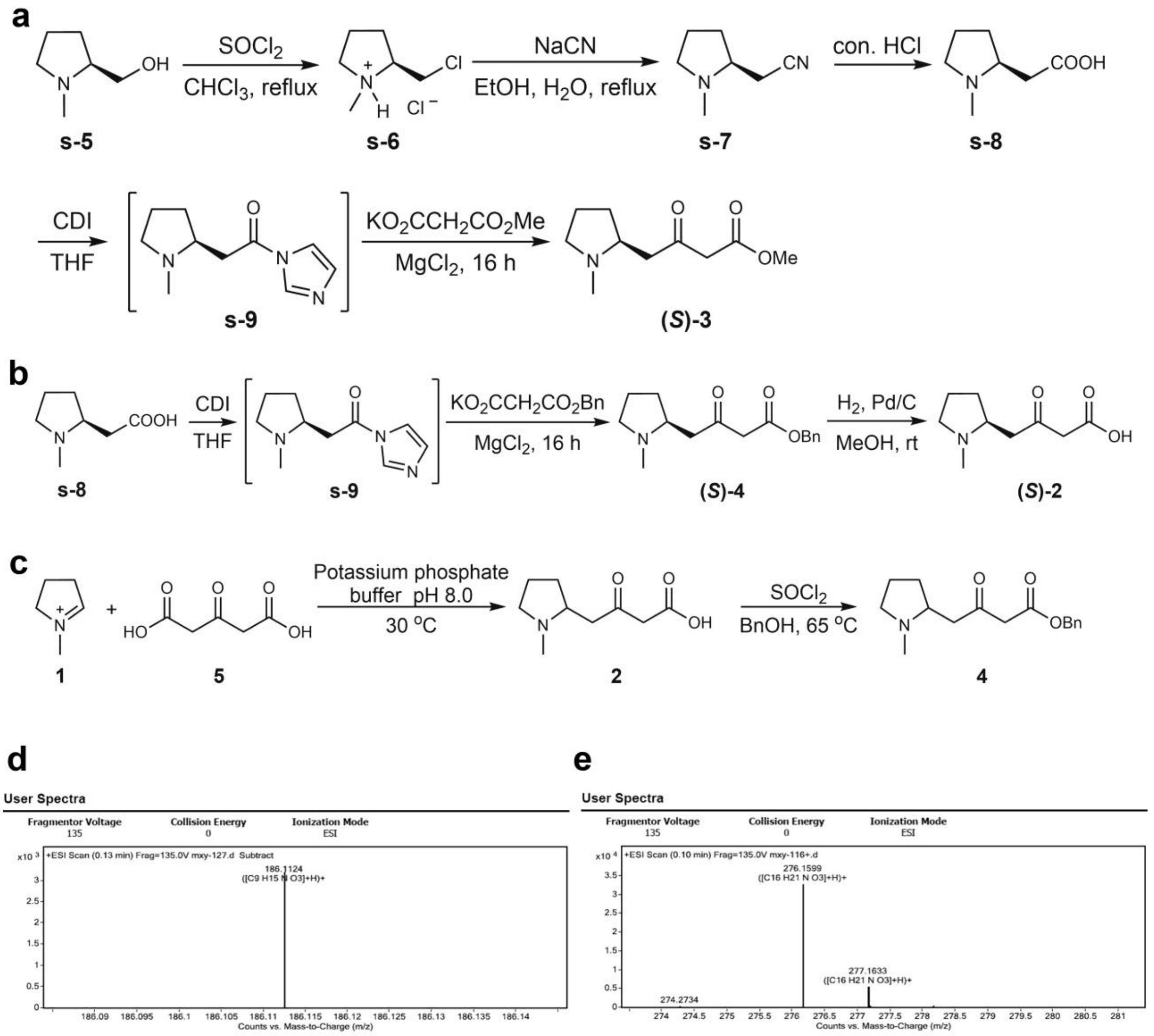

Supplementary Figure 7. Synthesis of chemical compounds 2, 3, and 4. a The synthetic route of methyl 4-(1-methylpyrrolidinyl)-3-oxobutanoate (**3**). **b** The synthetic route of 4-(1-methyl-2-pyrrolidinyl) 3-oxobutanoic acid (**2**) and benzyl 4-(1-methylpyrrolidinyl)-3-oxobutanoate (**4**). **c** The second synthetic route of 4-(1-methyl-2-pyrrolidinyl)3-oxobutanoic acid (**2**) and benzyl 4-(1-methylpyrrolidinyl)-3-oxobutanoate (**4**). **d** HRMS analysis of **2**. **e** HRMS analysis of **4**.

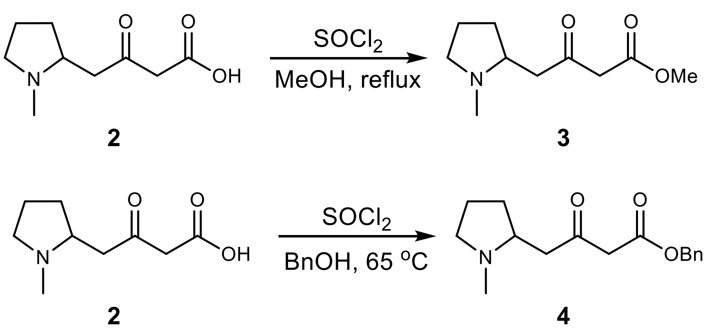

Supplementary Figure 8. Methyl and benzyl modification of the enzymatic product 2.

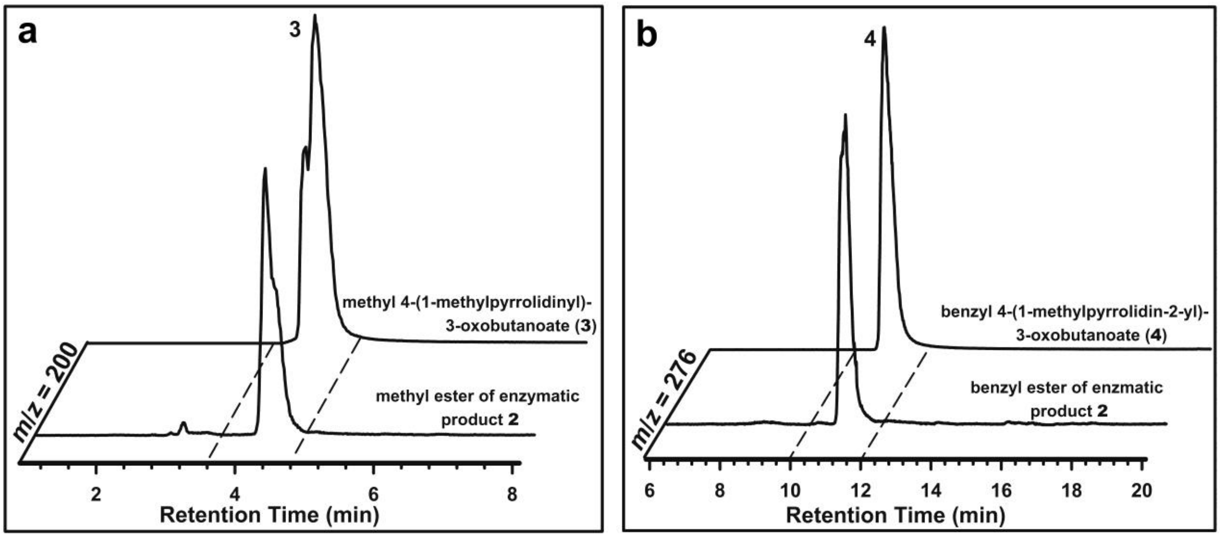

Supplementary Figure 9. LC-MS analysis of methyl or benzyl ester of enzymatic product 2. a LC-MS analysis of methyl ester of enzymatic product **2** ($[M + H]^+ = 200$). **b** LC-MS analysis of benzyl ester of enzymatic product **2** ($[M + H]^+ = 276$).

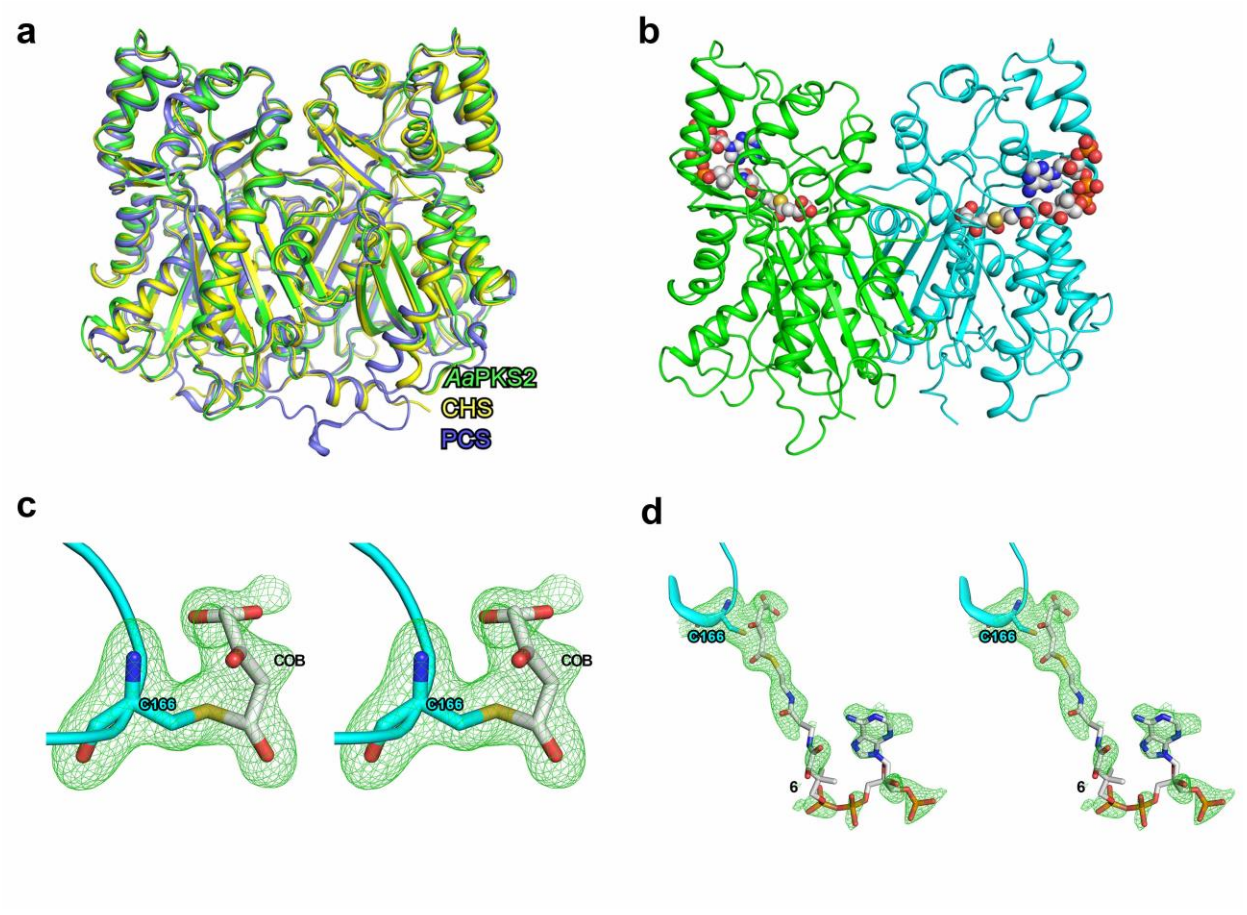

Supplementary Figure 10. The crystal structures of *Aa*PYKS-COB and *Aa*PYKS-6. a Superimposition of overall structures of *Aa*PYKS-COB (AaPKS2), *M. sativa* CHS (PDB: 1CML) and *A. arborescens* PCS (PDB: 2D3M). *Aa*PYKS, green; *Ms*CHS, yellow; *Aa*PCS, blue. **b** Overall structure of *Aa*PYKS-**6**. The molecules **6** are shown as spheres. **c** A stereo presentation of the Fo-Fc electron density polder map contoured at 5σ for the catalytic residue C166 and the covalently bound COB. **d**. A stereo presentation of the Fo-Fc electron density polder map contoured at 3σ for the catalytic residue C166 and **6**.

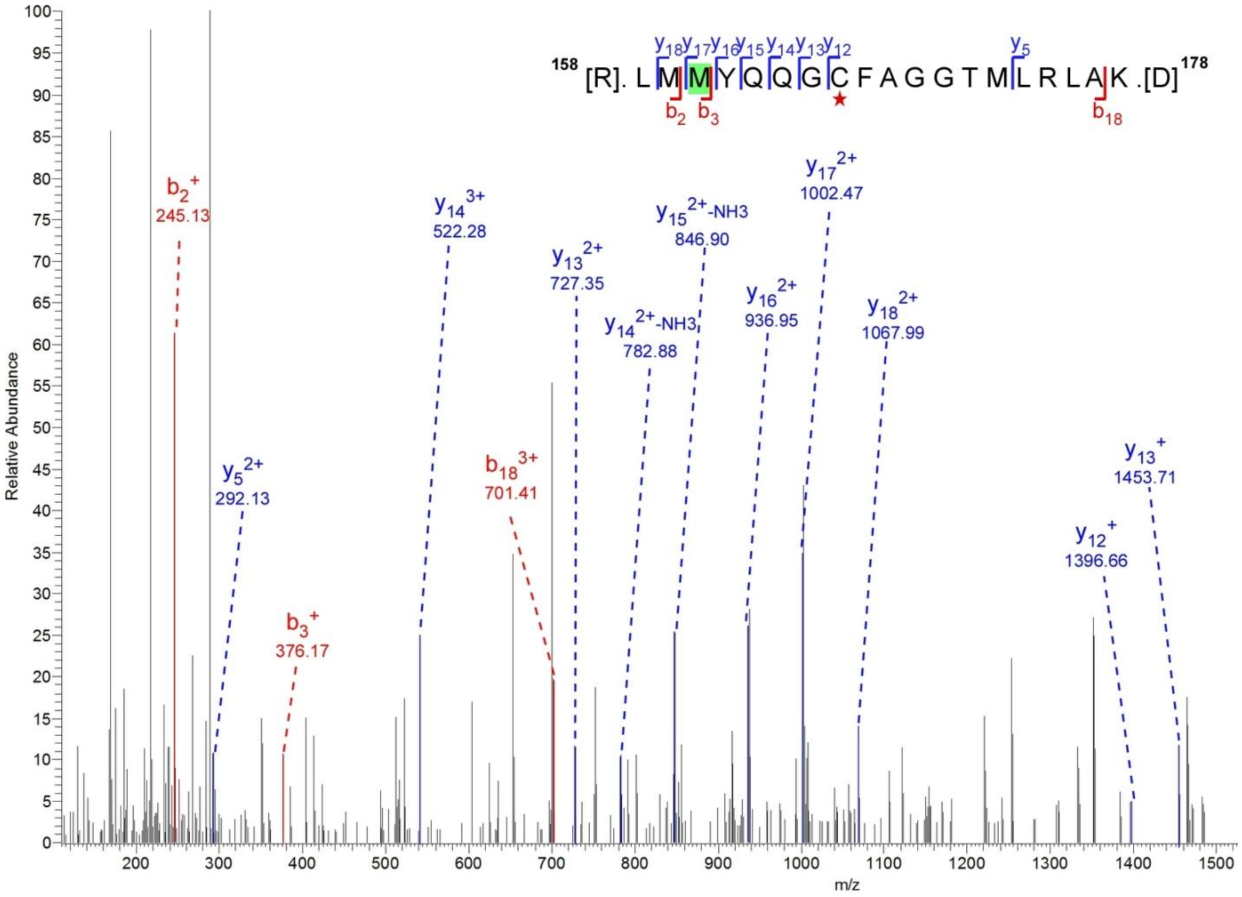

Supplementary Figure 11. **The characterization of covalent modification on C166 residue by an integrated HCD spectrum.** Red star indicates the modified catalytic residue C166. The oxidized Met161 during sample preparing is indicated by green.

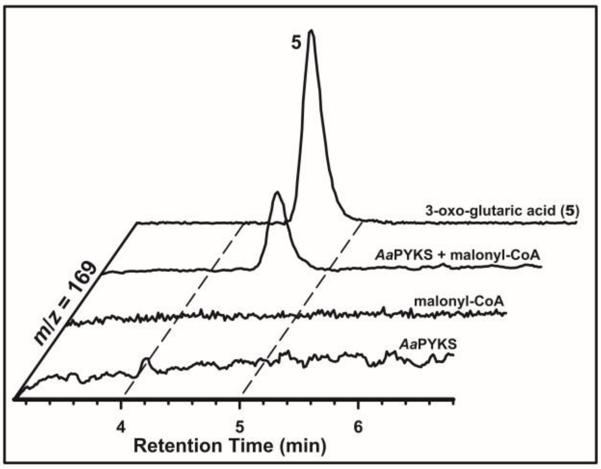

Supplementary Figure 12. **LC-MS analysis of the conversion of malonyl-CoA to 5 (*m/z* = 169, [M + Na]$^+$) by *Aa*PYKS in the absence of substrate 1.**

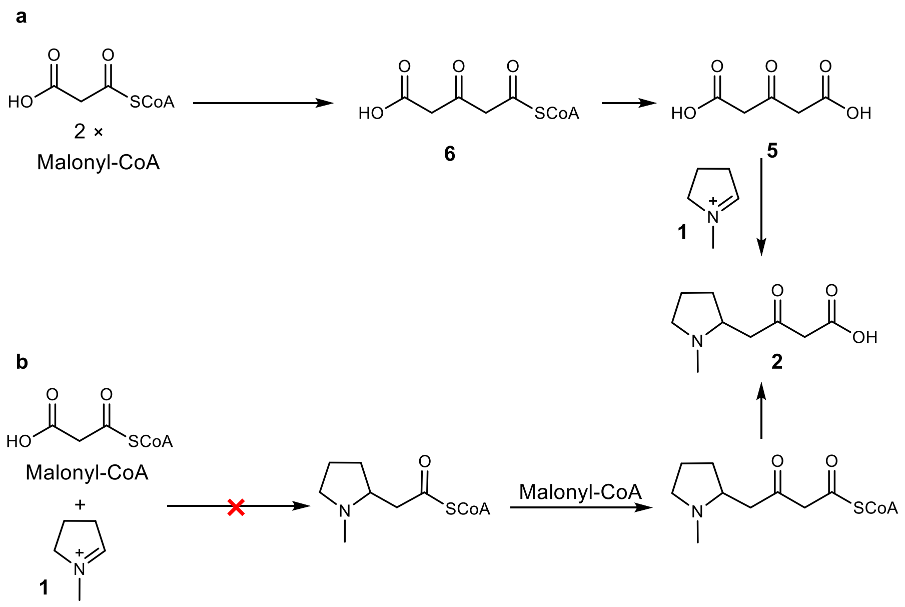

Supplementary Figure 13. The two different pathways for the formation of 2. a Two molecules of malonyl-CoA condensation first to generate the acid **5**. **b** Condensation between malonyl-CoA and **1** first.

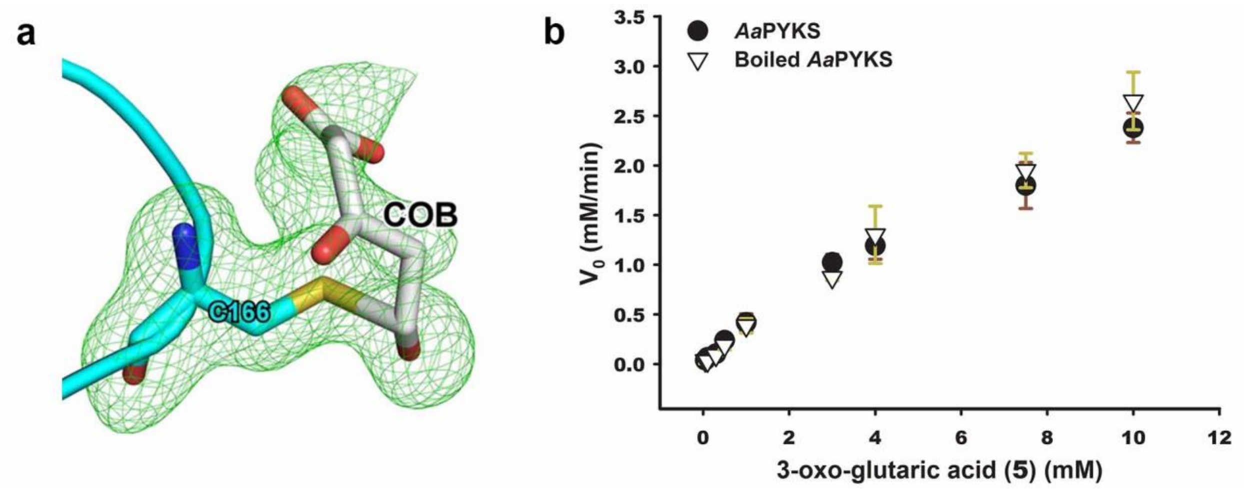

Supplementary Figure 14. Condensation 1 with 5 is a non-enzymatic reaction. a Soaking **1** into crystals of *Aa*PYKS-COB results no release of COB from C166 in the active center. Green mesh shows the F_O-F_C electron density polder map contoured at 5 σ of the catalytic residue C166 and the covalently bound COB. **b** Kinetic analysis demonstrating that the condensation **1** with **5** was non-enzymatic. Each reaction mixture initially contained 1mM **1**, and the indicated amounts of **5**. Values are means ± s.d. (*n*=2-3) from 3 technical replications.

.

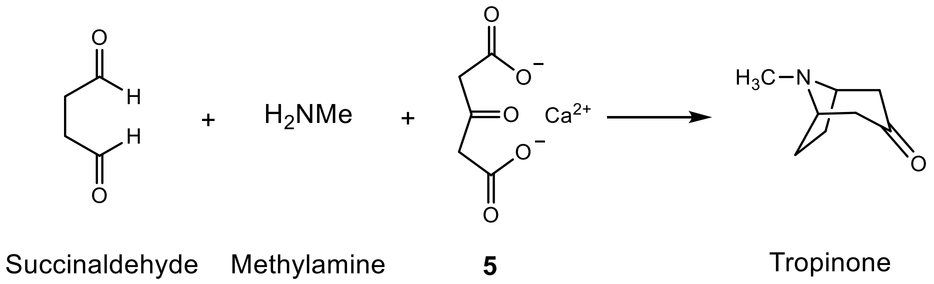

Supplementary Figure 15. The synthesis of tropinone published by Robert Robinson in 1917.

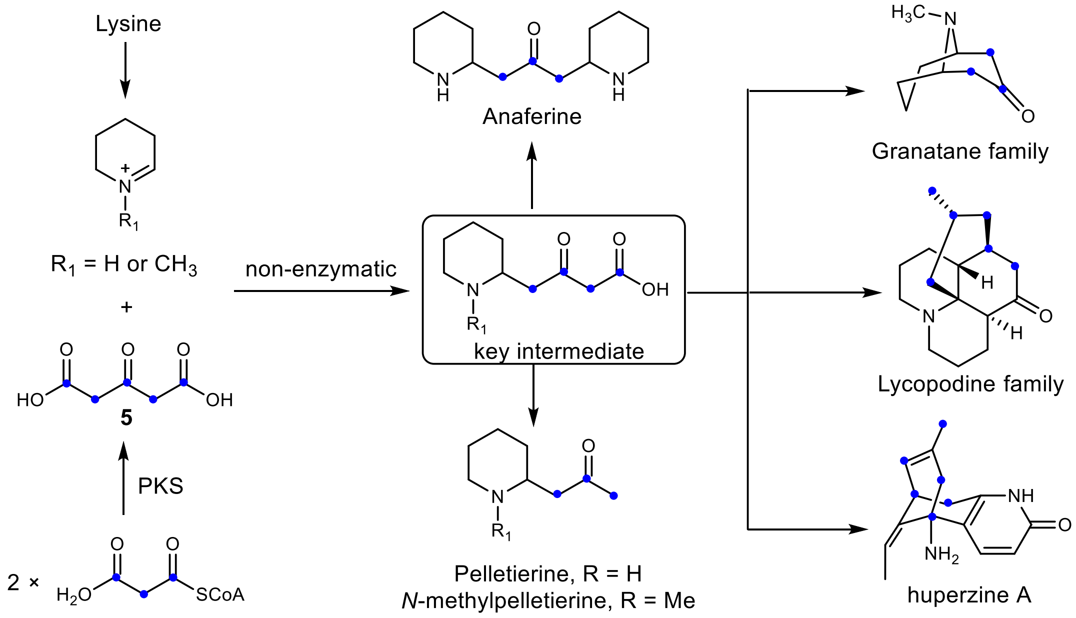

Supplementary Figure 16. Proposed biosynthetic pathway of GAs, pelletierine, anaferine, and lycopodium alkaloids via the key intermediate which is presumbaly synthesized by plant type III PKSs.

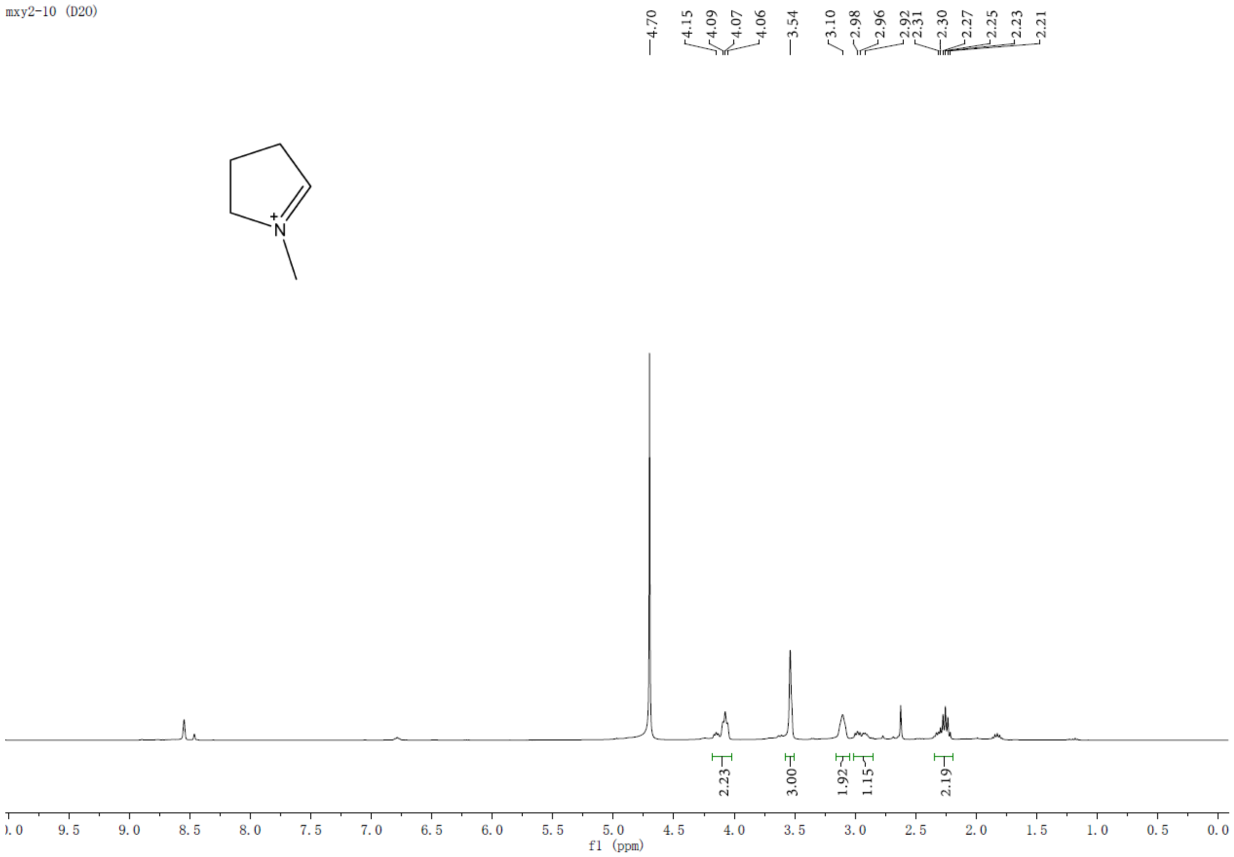

Supplementary Figure17. ^{1}H **NMR spectrum of compound 1 in** D_2O

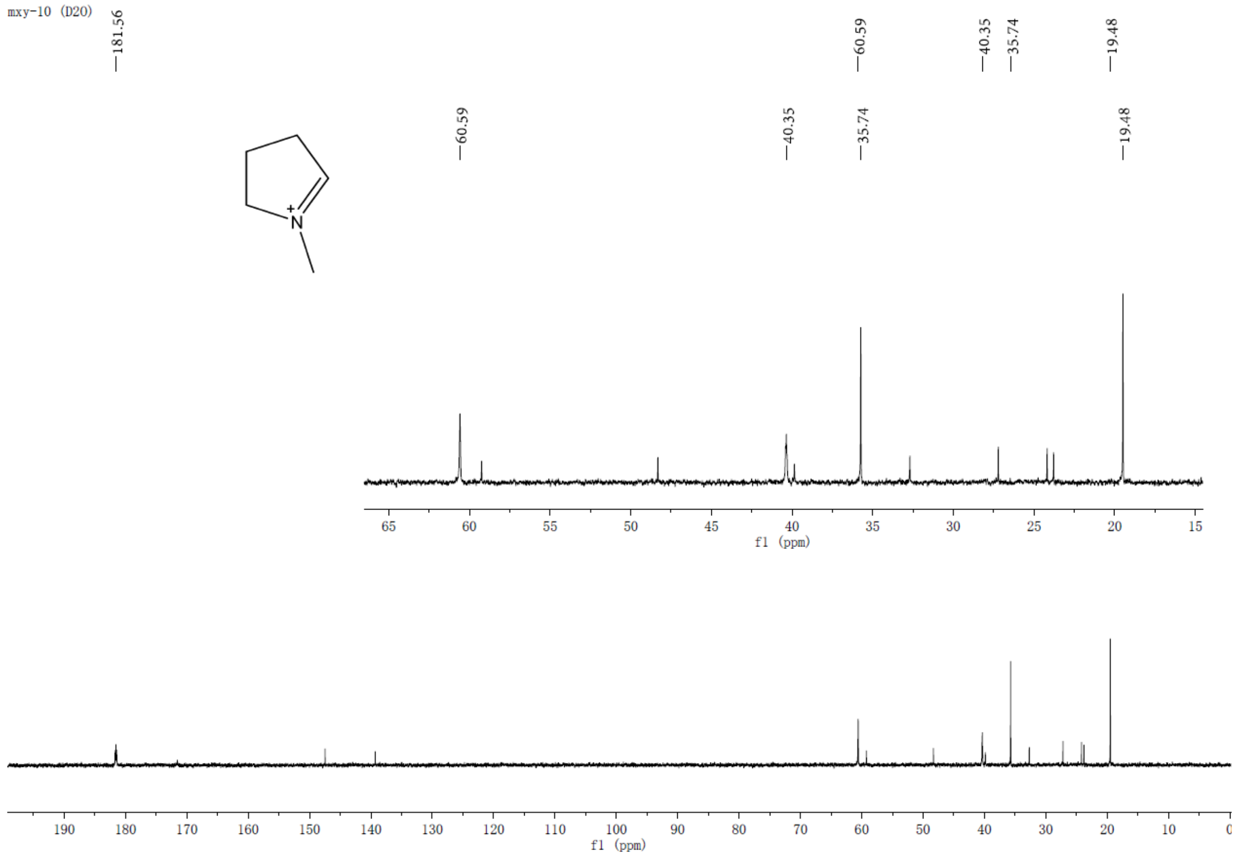

Supplementary Figure 18. ^{13}C **NMR spectrum of compound 1 in** D_2O

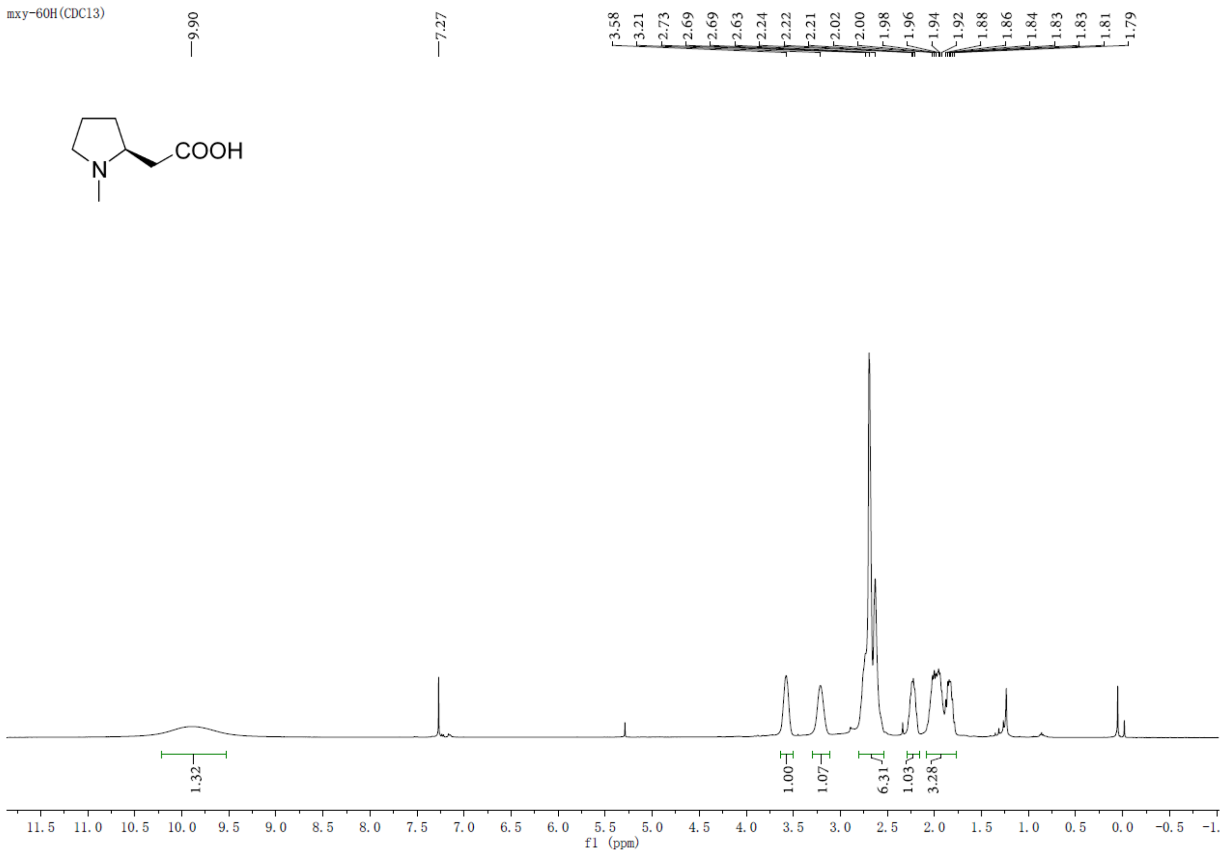

Supplementary Figure 19. **^{1}H NMR spectrum of s-8 in $CDCl_3$**

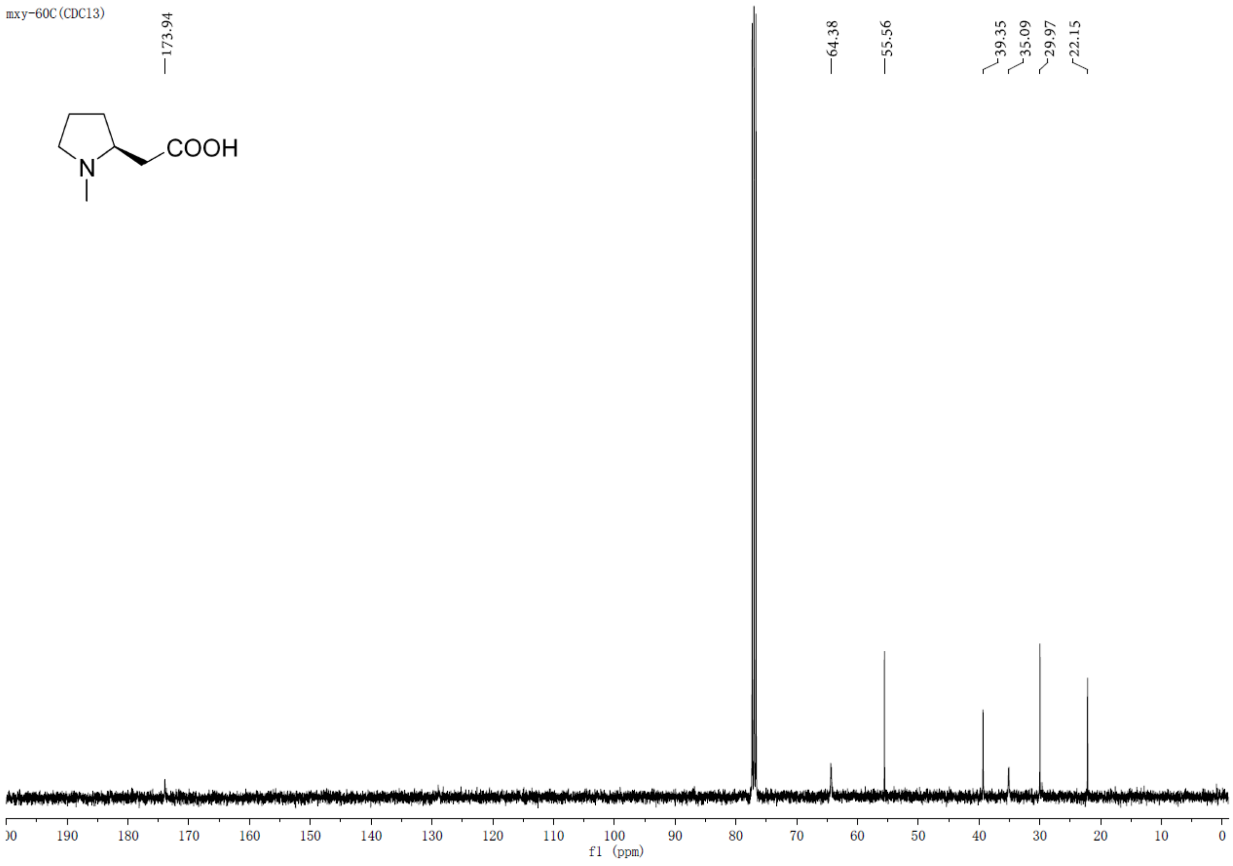

Supplementary Figure 20. **^{13}C NMR spectrum of s-8 in $CDCl_3$**

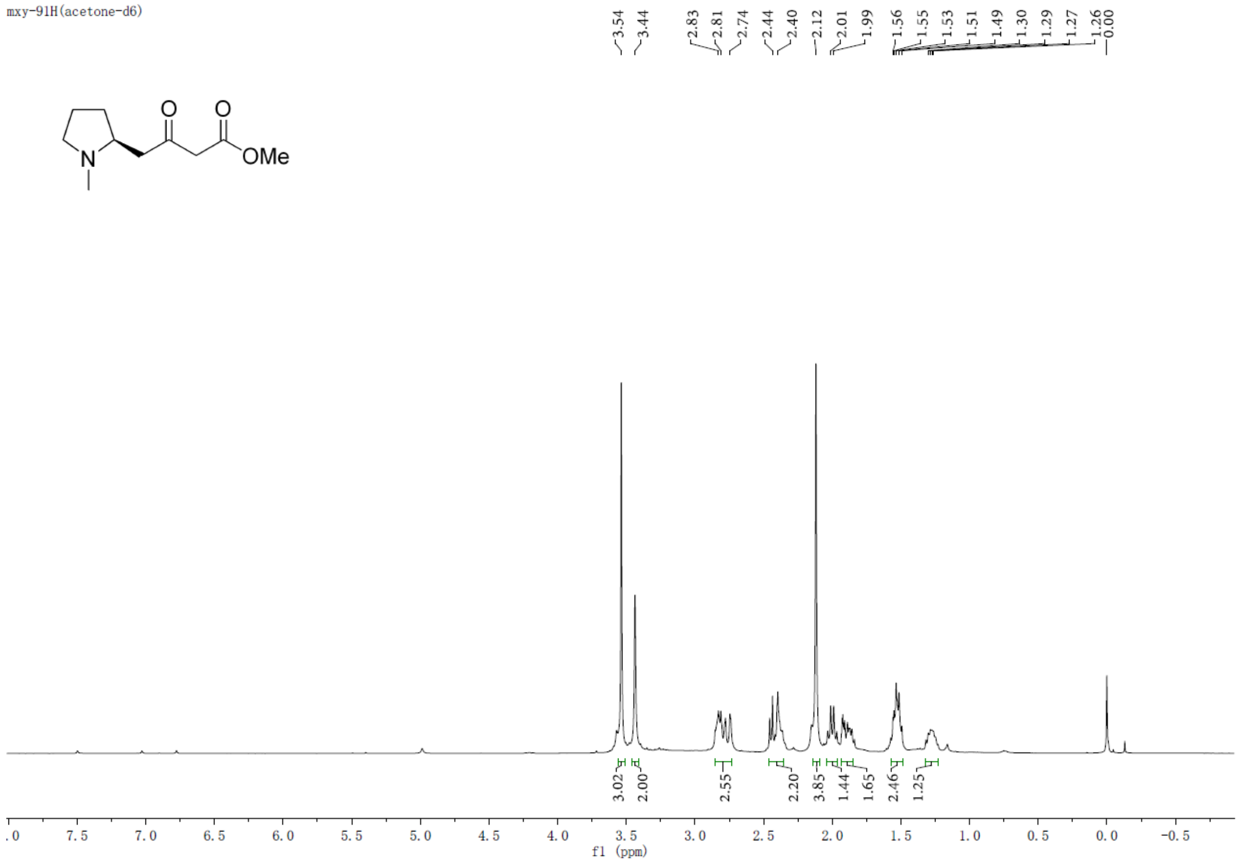

Supplementary Figure 21. ¹H NMR spectrum of (*S*)-3 in acetone-d_6

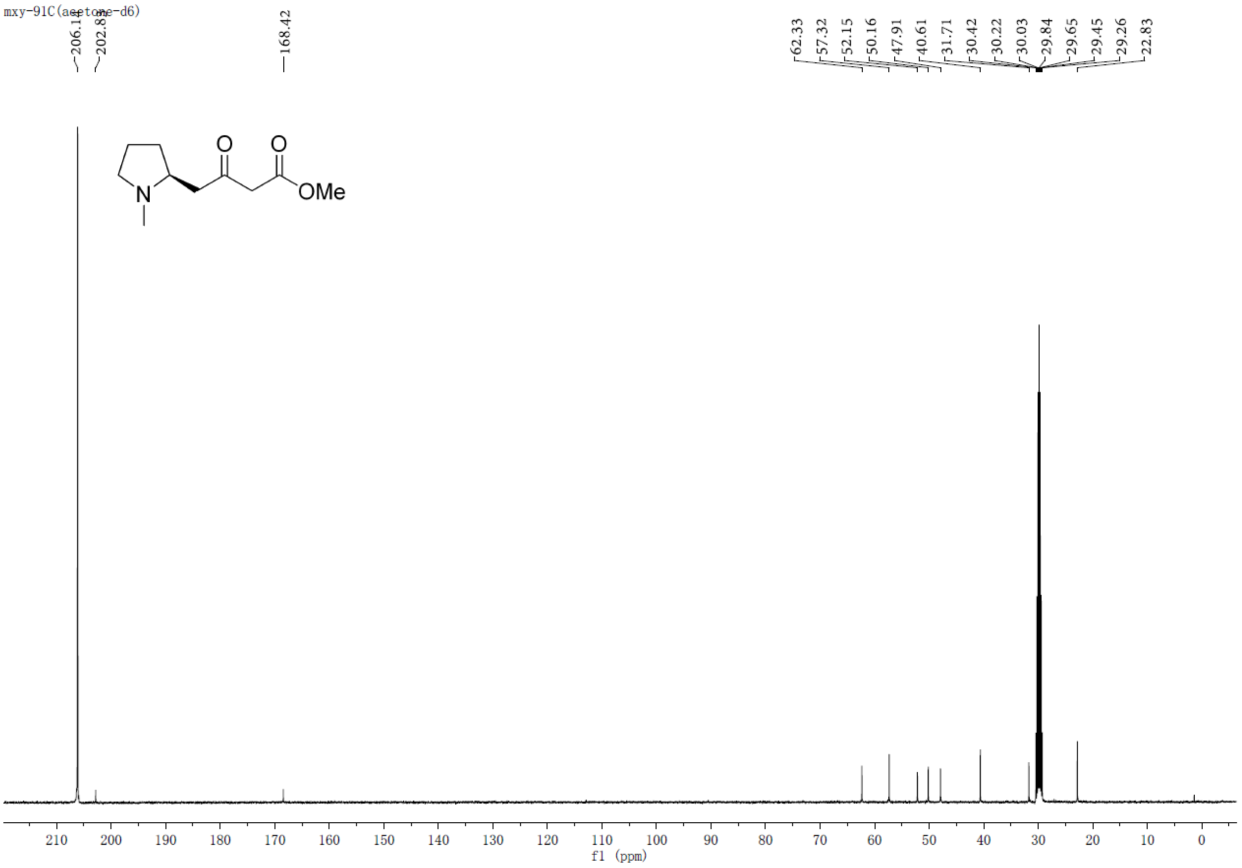

Supplementary Figure 22. ¹³C NMR spectrum of (*S*)-3 in acetone-d_6

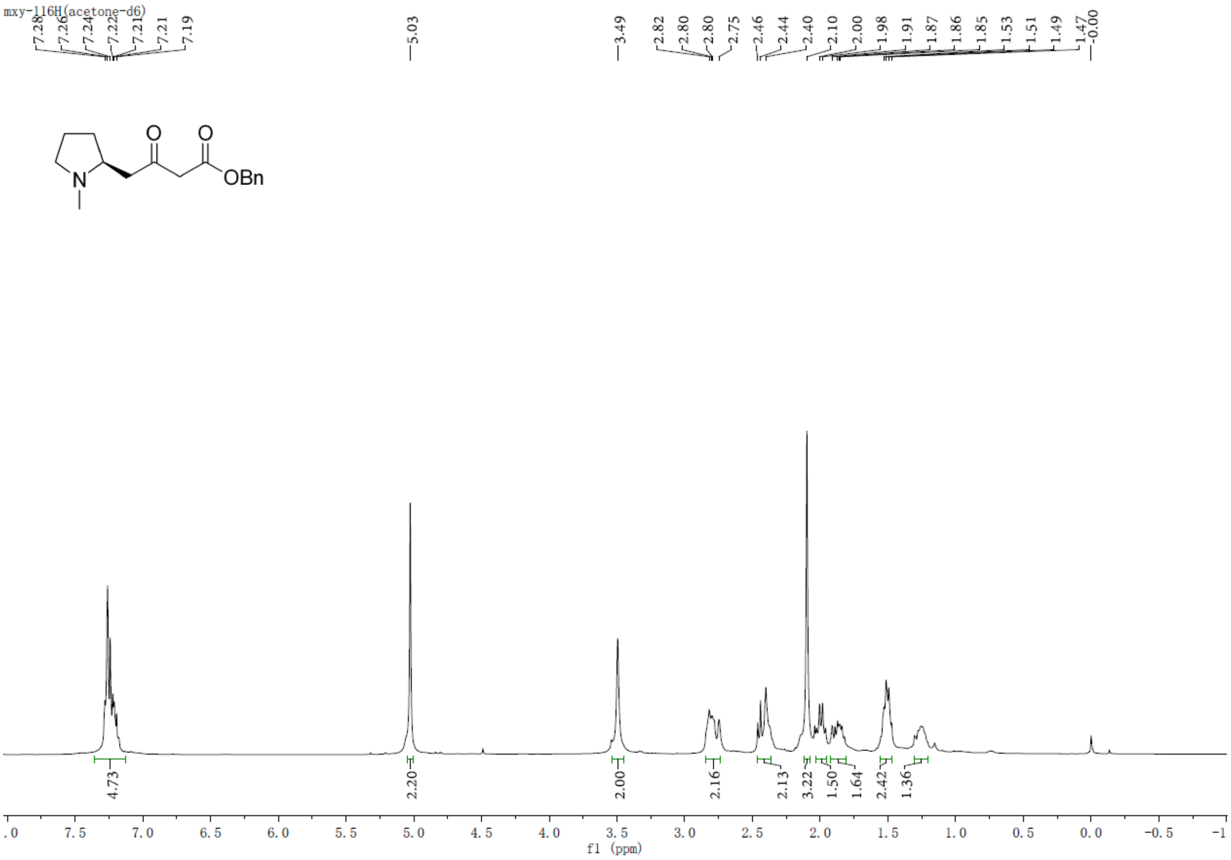

Supplementary Figure 23. **^{1}H NMR spectrum of (*S*)-4 in acetone-d_6**

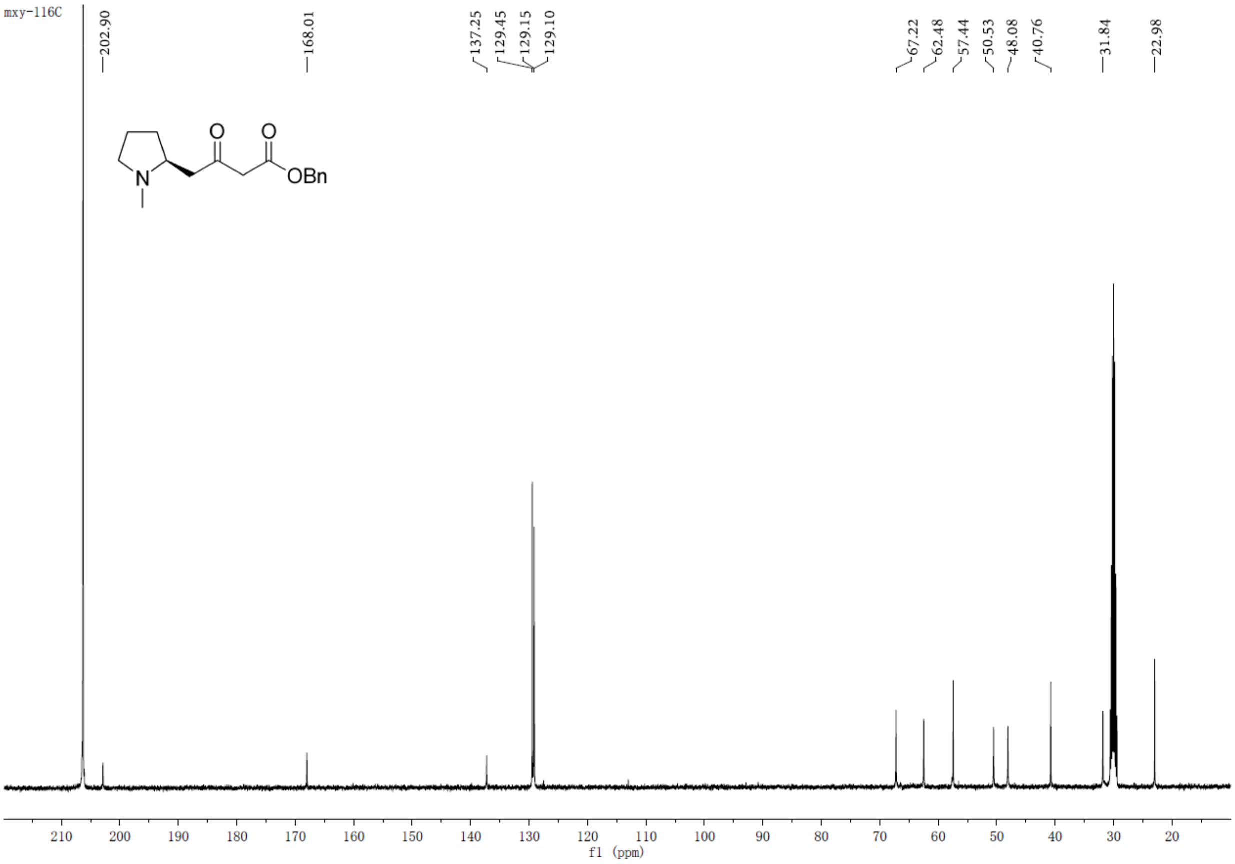

Supplementary Figure 24. **^{13}C NMR spectrum of (*S*)-4 in acetone-d_6**

Supplementary Table 1. **Plant type III polyketide synthase (PKS) unigenes obtained from hair root transcriptomes of *A. acutangulus, D. stramonium,* and *A. belladonna*.**

Unigene ID	Species	Gene Name	Full length	Protein
c187758_g1	*Anisodus acutangulus*	*AaPKS1*	1401 bp	43.10 KD
c2432_g1	*Anisodus acutangulus*	*AaPKS2*	1466 bp	42.85 KD
c76036_g1	*Anisodus acutangulus*	*AaPKS3*	1488 bp	34.63 KD
c92651_g1	*Anisodus acutangulus*	*AaPKS4*	1478 bp	42.37 KD
c18319_g1	*Atropa belladonna*	*AbPKS1*	909 bp	22.61 KD
c19217_g1	*Atropa belladonna*	*AbPKS2*	1242 bp	42.39 KD
c47080_g1	*Atropa belladonna*	*AbPKS3*	1424 bp	43.30 KD
c86521_g1	*Atropa belladonna*	*AbPKS4*	1233 bp	42.75 KD
c9024_g1	*Atropa belladonna*	*AbPKS5*	1426 bp	42.55 KD
c32476_g1	*Datura stramonium*	*DsPKS1*	1472 bp	43.13 KD
c9623_g1	*Datura stramonium*	*DsPKS2*	1252 bp	36.85 KD

Supplementary Table 2. Primers used in this study

Primer name	Purpose	Primers (5′-3′)
SMART II A	RACE-cDNA	AAGCAGTGGTATCAACGCAGAGTACGCGGG
3'-CDS	RACE-cDNA	AAGCAGTGGTATCAACGCAGAGTACTTTTTTTTTTTTTTTTTTTTTTTTTTTTTTVN
5'-CDS	RACE-cDNA	TTTTTTTTTTTTTTTTTTTTTTTTTVN
UPM Long	RACE-cDNA	CTAATACGACTCACTATAGGGCAAGCAGTGGTATCAACGCAGAGT
UPM Short	RACE-cDNA	CTAATACGACTCACTATAGGGC
NUP	RACE-cDNA	AAGCAGTGGTATCAACGCAGAGT
AbPKS1-F	3’ RACE	AGGAGAAGTTCAAGCGCATGTGTGT
AbPKS1-R	5’ RACE	CCCATTCATTGATGGCCTTTTGGGC
AbPKS4-F	3’ RACE	CCAACTGCGATGATCCGGAACTCAA
AbPKS4-R	5’ RACE	CGGATCATCGCAGTTGGTGTTT
AbPKS3-F	3’ RACE	GCCTAAGCCCATCAGTTCAACGTCT
AbPKS3-R	5’ RACE	ACACGACAAGTACCCTAGCTCCCTT
AaPKS3-F	3’ RACE	TGGCAGGCTTACTGAAGAGGG
AaPKS3-R	5’ RACE	CCTCCTGGTAATCTAGCTTCAC
AaPKS4-F	3’ RACE	CTCTTTTTGAGATTGTCACGGCGGC
AaPKS4-R	5’ RACE	GACGTTGAACTGATGGGCTTAG
DsPKS2-F	3’ RACE	GAGTGCTAGCAGAAGAGCTTTGGCA
DsPKS2-R	5’ RACE	GTGTCAGGCAAGAAATGCTG
DsPKS1-F	3’ RACE	CTCTTTTTGAGATTGTCACGGCGGC
DsPKS1-R	5’ RACE	CGCCGTGACAATCTCAAAAAGAGCC
AbPKS2-F	3’ RACE	GCGGCCGCTATAGTAGTTGGATCAG
AbPKS2-R	5’ RACE	AGGACTCGGGCCCTCTTATTGTTCT
AaPKS2-F	3’ RACE	TACTGGAGAAGGGCTGGACTTTGGT
AaPKS3-R	5’ RACE	GCCCTTCTCCAGTAGTTTTCAGCCC
AaPKS1-F	3’ RACE	GAGTGGGGCGTGCTTTGTAGTTTTG
AaPKS1-R	5’ RACE	GTCACTTAGGACATGCCTTGTAGC
AbPKS5-F	3’ RACE	GGCACGGTTTTAAGAATGGCCAAGG
AbPKS5-R	5’ RACE	ACTTCCACCACCACAATATCCTGCC
F-Aa2432-28a	pET28a-*AaPKS2*	CGGAATTC ATGAAGATGGGAAATGGTAAACA
R-Aa2432-28a	pET28a-*AaPKS2*	ACGCGTCGACACATTAAATGGGCTTACTATGGAGGA
F-Ab47080-28a	pET28a-*AbPKS3*	CGGGATCCATGAAGTTGGAAAATGGTCA

R-Ab47080-28a	pET28a-*AbPKS3*	ACGCGTCGACATGTTAAATGGGCACACTACG
F-Ds32476-28a	pET28a-*DsPKS1*	CGGGATCCATGAAGTTGGAAAATGGTCAAAA
R-Ds32476-28a	pET28a-*DsPKS1*	ACGCGTCGACTTATTAAATGGGCACACTGCGG
Aa134RA-F	pET28a-*AaPKS2R134A*	TTTAGTCTTTTGCACTGCAAGCGGCGTAGAC
Aa134RA-R	pET28a-*AaPKS2R134A*	GCAGTGCAAAAGACTAAATGGGTTATTGTG
Aa134RT-F	pET28a-*AaPKS2R134T*	TTTAGTCTTTTGCACTACAAGCGGCGTAGAC
Aa134RT-R	pET28a-*AaPKS2R134T*	GTAGTGCAAAAGACTAAATGGGTTATTGTG
Aa134RS-F	pET28a-*AaPKS2R134S*	TTTAGTCTTTTGCACTAGCAGCGGCGTAGAC
Aa134RS-R	pET28a-*AaPKS2R134S*	GCTAGTGCAAAAGACTAAATGGGTTATTGTG
Aa258LA-F	pET28a-*AaPKS2L258A*	GGGATTGTCACCTCGCGGCACACCTTCGC
Aa258LA-R	pET28a-*AaPKS2L258A*	*GCCGCGAGGTGACAATCCCCGTTAGGGAC*
Aa340SL-F	pET28a-*AaPKS2S40L*	TGACTTCGGGAATATGTTGAGTGCATGTG
Aa340SL-R	pET28a-*AaPKS2S40L*	AACATATTCCCGAAGTCACGAAGAATATT
Aa340SG-F	pET28a-*AaPKS2S40G*	GTGACTTCGGGAATATGGGGAGTGCATGTG
Aa340SG-R	pET28a-*AaPKS2S40G*	CCCATATTCCCGAAGTCACGAAGAATATT
Aa340SV-F	pET28a-*AaPKS2S40V*	GTGACTTCGGGAATATGGTGAGTGCATGTG
Aa340SV-R	pET28a-*AaPKS2S40V*	ACCATATTCCCGAAGTCACGAAGAATATT
