# Supplementary material for: Tropane alkaloids biosynthesis involves an unusual type III polyketide synthase and non-enzymatic condensation
Source: Nat Commun. 2019 Sep 6;10:4036. doi: 10.1038/s41467-019-11987-z (PMC6731253; doi:10.1038/s41467-019-11987-z)
Supplement: Supplementary file 2 — Reporting Summary [file 41467_2019_11987_MOESM2_ESM.pdf]

nature research

Corresponding author(s): Yu Zhang & Sheng-Xiong Huang

Last updated by author(s): Aug 5, 2019

Reporting Summary

Nature Research wishes to improve the reproducibility of the work that we publish. This form provides structure for consistency and transparency in reporting. For further information on Nature Research policies, see Authors & Referees and the Editorial Policy Checklist .

Statistics

For all statistical analyses, confirm that the following items are present in the figure legend, table legend, main text, or Methods section.

n/a	Confirmed	
☐	☒	The exact sample size (*n*) for each experimental group/condition, given as a discrete number and unit of measurement
☐	☒	A statement on whether measurements were taken from distinct samples or whether the same sample was measured repeatedly
☒	☐	The statistical test(s) used AND whether they are one- or two-sided<br>*Only common tests should be described solely by name; describe more complex techniques in the Methods section.*
☒	☐	A description of all covariates tested
☒	☐	A description of any assumptions or corrections, such as tests of normality and adjustment for multiple comparisons
☐	☒	A full description of the statistical parameters including central tendency (e.g. means) or other basic estimates (e.g. regression coefficient) AND variation (e.g. standard deviation) or associated estimates of uncertainty (e.g. confidence intervals)
☒	☐	For null hypothesis testing, the test statistic (e.g. *F*, *t*, *r*) with confidence intervals, effect sizes, degrees of freedom and *P* value noted<br>*Give P values as exact values whenever suitable.*
☒	☐	For Bayesian analysis, information on the choice of priors and Markov chain Monte Carlo settings
☒	☐	For hierarchical and complex designs, identification of the appropriate level for tests and full reporting of outcomes
☒	☐	Estimates of effect sizes (e.g. Cohen's *d*, Pearson's *r*), indicating how they were calculated

Our web collection on statistics for biologists contains articles on many of the points above.

Software and code

Policy information about availability of computer code

Data collection	Chromaster was used for HPLC analysis of TA alkaloids from hairy roots. Agilent MassHunter and LC/MSD ChemStation were used for enzymatic reaction analysis. MUSCLE implemented in MEGA version 7.0.14 was used for amino acid alignment, and GeneDoc version 2.7 was used for visualization. ChemBioDraw Ultra 14.0 was used for drawing chemical structures. HKL2000 was used to process protein crystal data.
Data analysis	SigmaPlot 12.0 was used for LC-MS data visualization. Adobe Photoshop CC 2015 and ChemBioDraw Ultra 14.0 were used for image editing. Coot and Phenix were used to perform protein structure model building. Proteome Discoverer 2.1 software was used to process protein LC-MS/MS data.

For manuscripts utilizing custom algorithms or software that are central to the research but not yet described in published literature, software must be made available to editors/reviewers. We strongly encourage code deposition in a community repository (e.g. GitHub). See the Nature Research guidelines for submitting code & software for further information.

Data

Policy information about availability of data

All manuscripts must include a data availability statement. This statement should provide the following information, where applicable:

- Accession codes, unique identifiers, or web links for publicly available datasets
- A list of figures that have associated raw data
- A description of any restrictions on data availability

RNA-Seq data that support the findings of this study have been deposited in National Center for Biotechnology Information (NCBI) Sequence Read Archive (SRA) with accession number SRR9888534, SRR9888536, and SRR9888538. The GenBank accession numbers for AaPYKS, DsPYKS, and AbPKS2 are MN025472, MN025473, and MN025474, respectively. The PDB IDs for AaPYKS-COB and AaPYKS-6 are 6J1M and 6J1N, respectively. The authors declare that all other relevant data supporting the findings of this study are available within the article, its Supplementary Information and Source Data files.

Field-specific reporting

Please select the one below that is the best fit for your research. If you are not sure, read the appropriate sections before making your selection.

- [x] Life sciences
- [ ] Behavioural & social sciences
- [ ] Ecological, evolutionary & environmental sciences

For a reference copy of the document with all sections, see nature.com/documents/nr-reporting-summary-flat.pdf

Life sciences study design

All studies must disclose on these points even when the disclosure is negative.

Sample size	The sample size has been described in the figure legends.
Data exclusions	No data was excluded.
Replication	The enzyme activity was analyzed with at least three biological replicates. The non-enzymatic condensation 1 with 5 was analyzed with three technical replicates.
Randomization	The three plant species chosen for this study were based on their availability, TA production, and the family and genus they belong to.
Blinding	The study material is plant, and the gene clone, protein expression, enzyme reaction were analyzed by instruments.

Reporting for specific materials, systems and methods

We require information from authors about some types of materials, experimental systems and methods used in many studies. Here, indicate whether each material, system or method listed is relevant to your study. If you are not sure if a list item applies to your research, read the appropriate section before selecting a response.

Materials & experimental systems

n/a	Involved in the study	
x		Antibodies
x		Eukaryotic cell lines
x		Palaeontology
	x	Animals and other organisms
x		Human research participants
x		Clinical data

Methods

n/a	Involved in the study	
x		ChIP-seq
x		Flow cytometry
x		MRI-based neuroimaging

Animals and other organisms

Policy information about studies involving animals; ARRIVE guidelines recommended for reporting animal research

Laboratory animals	It is not relevant to this study.
Wild animals	It is not relevant to this study.
Field-collected samples	The study did not involve samples collected from the field.
Ethics oversight	No ethical approval or guidance was required, because the organisms we used in this study are regular plants cultured in the lab.

Note that full information on the approval of the study protocol must also be provided in the manuscript.
